# Magnetic field promoted irreversible process of water based nanocomposites with heat and mass transfer flow

**DOI:** 10.1038/s41598-020-80554-0

**Published:** 2021-01-18

**Authors:** Noor Saeed Khan, Poom Kumam, Phatiphat Thounthong

**Affiliations:** 1 KMUTTFixed Point Research Laboratory, Room SCL 802 Fixed Point Laboratory, Science Laboratory Building, Department of Mathematics, Faculty of Science, King Mongkut’s University of Technology Thonburi (KMUTT), Bangkok, 10140 Thailand; 2 Center of Excellence in Theoretical and Computational Science (TaCS-CoE), Science Laboratory Building, Faculty of Science, King Mongkut’s University of Technology Thonburi (KMUTT), 126 Pracha-Uthit Road, Bang Mod, Thrung Khru, Bangkok 10140 Thailand; 3 Department of Medical Research, China Medical University Hospital, China Medical University, Taichung, 40402 Taiwan; 4 Department of Mathematics, Division of Science and Technology, University of Education, Lahore, 54000 Pakistan; 5 Renewable Energy Research Centre, Department of Teacher Training in Electrical Engineering, Faculty of Technical Education, King Mongkut’s University of Technology North Bangkok, 1518 Pracharat 1 Road, Wongsawang, Bangsue, Bangkok 10800 Thailand

**Keywords:** Engineering, Materials science, Mathematics and computing, Nanoscience and technology, Physics

## Abstract

Analytical analysis of two-dimensional, magnetohydrodynamic, heat and mass transfer flow of hybrid nanofluid incorporating Hall and ion-slip effects and viscous dissipation in the presence of homogeneous-heterogeneous chemical reactions and entropy generation is performed. The governing equations are modified with the help of similarity variables. The reduced resulting nonlinear coupled ordinary differential equations are solved with the help of homotopy analysis method. The effects of all the physical parameters are demonstrated graphically through a detailed analysis. The main outcome of the study is the use of applied strong magnetic field which generates the cross flow of hybrid nanofluid along the *z*-axis. The numerical comparison to the existing published literature is also provided.

## Introduction

Magnetism has revolutionized a wide spectrum of technologies such as data storage and biomedical imaging and continues to bring forth new phenomena in emergent materials of reduced dimensionalities. In 2017, magnetic 2D materials emerged as ideal solid-state platforms where both crystalline structural order and long-range magnetic order coexist and couple in atomic-thin region, which makes the unprecedented experimental explorations of 2D magnetism routinely feasible in numerous laboratories worldwide. Furthermore, the seamless integration of 2D magnets with dissimilar electronic and photonic 2D materials, backed by the already mature techniques on high-quality van der Waals heterostructure constructions, opens up remarkable opportunities for a plethora of designer quantum heterostructures with previously inaccessible magnetoelectric and magneto-optical properties.

Scientists and engineers are devoted to research perspectives and reports on recent advances in material innovations on new 2D magnets, magneto-optical, magnetoelectric, and a variety of nanoscale characterizations of 2D magnetic systems, and theoretical prediction and understanding of novel 2D magnetic, spintronic, and magnonic properties, as well as exotic magnetism in relevant systems including twisted bilayer graphene, topological insulators, and Weyl semimetals. The addition of 2D magnets greatly expands the family of 2D materials, and the advanced spintronic devices they enable could reshape the landscape of nanoelectronics and nanospintronics in miniaturized form factors. Mahdy et al.^1^ established a mathematical analysis for the magnetohydrodynamics, homogeneous-heterogeneous chemical reactions in flow resulted by an impulsively rotating sphere. Using finite difference technique in combination with quasi-linearization scheme for the solution, they proved that magnetic field reduces the velocity components due to resistive type Lorentz force. Basha et al.^2^ applied the Runge-Kutta fourth order integration scheme with shooting technique to compute the solution for magnetohydrodynamic boundary layer flow of viscous incompressible fluid with leading edge accretion and ablation. Ilias et al.^3^ studied theoretically the unsteady aligned magnetohydrodynamic boundary layer flow and heat transfer of magnetite and alumina nanoparticles through a vertical flate plate with leading edge accretion. Mabood and Khan^4^ investigated the effects of magnetic field on Blasius and Sakiadis flow with leading edge accretion and ablation whose most relevant outcome is that for the magnetic field strength the thinner boundary layer is established in Blasius and Sakiadis flows.

For decades, the field of thermoelectricity has been dominated by inorganic materials. The demonstration of reasonably stable polymer-based materials with a figure of merit of 0.1 and above has triggered considerable interest in organic and hybrid thermoelectric materials during the last 10 years. Materials of current interest include both small-molecule and polymeric semiconductors, carbonaceous nanoparticles such as carbon nanotubes, graphene and graphene oxide, as well as composite materials of organic or inorganic nanoparticles embedded in a polymer matrix. However, these materials pose numerous fundamental challenges with regard to their thermoelectric properties in relation to processing and doping, molecular and solid-state structures as well as electronic properties. Key aspects of current interest for the community concern (1) the synthesis of tailor-made materials, (2) doping mechanisms of both n- and p-type materials, (3) the interplay of the rich nano- and microstructure with charge and heat transport, as well as (4) the environmental and thermal stability of novel materials. New device architectures and device concepts such as flexible thermoelectric generators printed on plastic foils or integrated into textile substrates are now accessible thanks to the inherent mechanical robustness of polymers. This special topic issue will bring together the scientific sectors in the field and assess recent progress in organic and hybrid thermoelectrics. Tabasum et al.^5^ offered a mathematical study of oblique transport of titanium water-based nano-polymer gels with mixed convection effects to simulate the real nano-polymer boundary interface dynamics, convective surface along with velocity slip. Sheikholeslami et al.^6^ reported the aluminum oxide-water nanofluid free convection due to Lorentz forces through a porous cubic domain incorporating ellipse shaped obstacle by employing the Lattice Boltzmann method. Pourmehran et al.^7^ used a semi exact method to compute the governing nonlinear coupled equations of nanofluid flow and heat transfer which shows that the least square method is suitable for computational work. Sheikholeslami et al.^8^ examined numerically the hydrothermal analysis of nanofluid during solidification using time dependent mesh. Mehmood et al.^9^ employed the scaling group of transformations to reduce the governing equations of crosswise stream of hydrogen-oxide through a porous media immersed with copper nanoparticles. AbdRabbuh et al.^10^ experimentally investigated the performance of heat transfer, thermophysical properties and pressure drop of GNP-based water nanofluid in the different configurations of heat exchanger tubes. Pourmehran et al.^11^ simulated the Patel model for the nanofluid flow and heat transfer between two contracting and rotating disks by investigating different types of nanoparticles. Samsudin et al.^12^ elucidated the characteristics of cellulose nanoparticles extracted from microcrystalline cellulose by hydrolysis reaction using 1-butyl-3-methylimidazolium acetate as a catalyst and solvent at various temperatures. Abdelaziz^13^ worked on the steady boundary layer magnetohydrodynamic slip flow past a stretching sheet in a water based nanofluid in the presence of Hall effect. Biglarian et al.^14^ investigated numerically the problem of unsteady magnetohydrodynamic nanofluid flow and heat transfer between parallel plates using fourth-order Runge-Kutta method. Pourmehran et al.^15^ worked on the Forchheimer-Brinkman-extended equation for the thermodynamics of a fin shaped microchannel heat sink cooled by different nanofluids. They used th KKL correlation for the calculation of effective thermal conductivity and viscosity and applied the central composite design to obtain the desirability of the optimum value of the nanofluid flow characteristics. Adesanya et al.^16^ investigated the entropy generation in a gravity aided thin couple stress liquid film in an inclined heated substrate whose results show that slip and porosity parameters are turns out to be large at the free surface of the porous substrate. Seikh et al.^17^ presented the effects of nanoparticles and uniform magnetic field on the slip blood flow conveyed through the hollow arterial tube as a third-grade fluid. Dutta et al.^18^ considered the entropy generation in a human respiratory tract model with realistic length to diameter ratio at different branches for the body temperature 36$^{\circ }$C taken into account the ambient condition of air at 25 $^{\circ }$C DBT and 50% RH. Other studies on nanofluids can be seen in the references^19–25^.

In recent years, hybrid nanocomposite materials have been considered as new generation of enhanced performance objects due to their unique characteristics emerging from the combination of hybrid materials. Hybrid nanofluids present the outstanding properties of rigidity, excellent thermal stability and improved mechanical strength. Akermi et al.^26^ investigated the effects nanoparticle concentration on the efficiency of hybrid polymer thin layers for studying the morphology, the structure and the optical behavior of the different synthesized nanomaterials, FTIR transmission spectra, scanning electron microscopy, transmission electron microscopy, and X-ray diffractometry. Waini et al.^27^ examined the stagnation point flow of hybrid nanofluid containing copper and alumina in water based fluid. They used the BVP4c, available in Matlab software to notice that the bifurcation of the solutions occurs in the shrinking regions and between the two solutions, in which only one of them is stable. Sreedevi et al.^28^ analyzed the unsteady, magnetohydrodynamic, heat and mass transfer hybrid nanofluid flow past a stretching surface with thermal radiation, chemical reaction, suction and slip effects. Waini et al.^29^ reported the squeezed hybrid nanofluid flow past a permeable sensor surface for numerical solutions in which dual solutions exist for some values of the porosity parameter. They found that the thermal conductivity was greater for the hybrid nanofluid, compared to the regular nanofluid. Afridi et al.^30^ carried out an analysis of the mono nanofluid and hybrid nanofluid with the entropy generation past a thin needle under the modified Maxwell Garnet and the Brinkman model using Runge-Kutta Fehlberg scheme. Hybrid nanofluids literature can also seen in the references^31–33^.

Entropy generation is associated with the irreversible process of any thermodynamics system. Bhatti et al.^34^ investigated the entropy generation past a moving surface with thermal radiation and chemical reaction using successive linearization method and chebyshev spectral collocation method for the reduced resulting non-linear coupled ordinary differential equations. Farooq et al.^35^ presented the entropy analysis of three-dimensional bioconvection flow of nanofluid past a linearly moving plate in the presence of magnetic field and microorganisms by applying the BVP4c MATLAB algorithm. Mondal et al.^36^ discussed the entropy generation and provided the dual solutions with the spectral quasiliearization method for the magnetohydrodynamic three-dimensional convective flow and heat transfer in a nanofluid. Afridi and Qasim^37^ performed the theoretical analysis of entropy generation of three-dimensional boundary layer flow past a bidirectional exponentially stretching surface. Relevant studies about entropy generation can also be seen in the references^38–40^.

This research presents the modeling and computing dynamics of a wide range of applications, from heat transferring devices and chaotic behavior of power systems, to the design of a chemical reactions network aimed for synchronous the present system control whose solution is accomplished through HAM^41^.

## Methods

**Figure 1:**
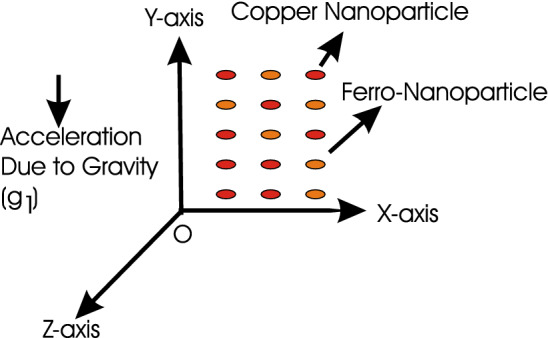
Schematic diagram of the problem.

An incompressible viscous fluid flow is investigated with free stream velocity $U_{\infty }$, homogeneous-heterogeneous chemical reactions and temperature *T*. In water base fluid, two types of nanoparticles namely $\hbox {Fe}_{3}\hbox {O}_{4}$ (magnetite) and Cu (copper) are considered respectively. Magnetic field ${\varvec{B}} = [0, B_{0}, 0]$ is applied perpendicularly to the heat and mass transfer flow. Assuming that the strength of electric charge and magnetic field are maximum. The schematic diagram of the problem is shown in Fig. 1. The problem has the following governing equations for hybrid nanofluid as in^1–4,13^

1 $$\begin{aligned}{}&\frac{\partial u}{\partial x} + \frac{\partial v}{\partial y} = 0, \end{aligned}$$

2 $$\begin{aligned}{}&\frac{\partial u}{\partial t} + \textit{u}\frac{\partial u}{\partial x} + \textit{v}\frac{\partial u}{\partial y} = {U_{\infty }}\frac{{\partial U_{\infty }}}{\partial x} + \frac{\mu _{hnf}}{\rho _{hnf}}\frac{\partial ^{2}{} \textit{u}}{\partial y^{2}} - \frac{\sigma _{hnf}{B^{2}_{0}}[(1 + m_{1}m_{2})u + m_{1}w]}{\rho _{hnf}[(1 + m_{1}m_{2})^{2} + m_{1}^{2}]} + \beta _{T}g_{1}(T - T_{\infty }) , \end{aligned}$$

3 $$\begin{aligned}{}&\frac{\partial w}{\partial t} + \textit{u}\frac{\partial w}{\partial x} + \textit{v}\frac{\partial w}{\partial y} = {U_{\infty }}\frac{{\partial U_{\infty }}}{\partial x} + \frac{\mu _{hnf}}{\rho _{hnf}}\frac{\partial ^{2}{} \textit{w}}{\partial {y^{2}}} + \frac{\sigma _{hnf}{B^{2}_{0}}[m_{1}u - (1 + m_{1}m_{2})w]}{\rho _{hnf}[(1 + m_{1}m_{2})^{2} + m_{1}^{2}]}, \end{aligned}$$

4 $$\begin{aligned}{}&\frac{\partial T}{\partial t} + {\textit{u}}\frac{\partial T}{\partial x} + \textit{v}\frac{\partial T}{\partial y} = \frac{k_{hnf}}{(\rho c_{P})_{hnf}}\frac{\partial ^{2}{} \textit{T}}{\partial {y^{2}}} + \frac{\mu _{hnf}}{(\rho c_{P})_{hnf}}\left[\frac{\partial \textit{u}}{\partial \textit{y}}\right]^{2}, \end{aligned}$$

5 $$\begin{aligned}{}&\frac{\partial b}{\partial t} + u \frac{\partial b}{\partial x} + v \frac{\partial b}{\partial y} = {D_{B}} \left(\frac{\partial ^{2} b}{\partial y^{2}} \right) - {k_{c}}{} \textit{b}{c^{2}}, \end{aligned}$$

6 $$\begin{aligned}{}&\frac{\partial c}{\partial t} + u \frac{\partial c}{\partial x} + v \frac{\partial c}{\partial y} = {D_{C}} \left(\frac{\partial ^{2} c}{\partial y^{2}} \right) + {k_{c}}{} \textit{b}{c^{2}}, \end{aligned}$$

where *u*, *v*, *w* are the velocity components in *x*, *y*, *z* directions, $m_{1} = \omega _{e}\tau _{e}$ and $m_{2} = \frac{en_{e}B_{0}}{(1 + n_{e}/n_{a})}$*Fr* are the Hall and ion-slip parameters in which *e*, $\omega _{e}$, $\tau _{e}$, $n_{e}$, $n_{a}$ and *Fr* are the electron, electron frequency, electron collision time, electron number density, neutral particle density and friction coefficient between ions and neutral particles. $\beta _{T}$ is the coefficient of thermal expansion. *B* and *C* are chemical species having concentrations *b* and *c* respectively while *D* is diffusion of species. Also $k_{c}$ and $k_{s}$ manifest the rate constant. The subscript hnf denotes the hybrid nanofluid. $\mu _{hnf}$, $\rho _{hnf}$, $k_{hnf}$ and $(\rho c_{P})_{hnf}$ are respectively the dynamic viscosity, density, thermal conductivity and heat capacity of hybrid nanofluid.

Boundary conditions are used as

7 $$\begin{aligned}{}&{u} = {u_{w}} = \lambda {U_{\infty }},\quad {v} = 0,\quad {w} = 0,\quad {T} = {T _{w} }, \quad {D_{B}}\frac{\partial b}{\partial y} = {k_{s}}{b}, \quad {D_{C}}\frac{\partial c}{\partial y} = - {k_{s}}{b}, \quad at \quad {y} = 0, \end{aligned}$$

8 $$\begin{aligned}{}&{u} = {u_{e}} = {U_{\infty }},\quad {w} \rightarrow 0, \quad {T} \rightarrow {T}_{\infty }, \quad \textit{b}\rightarrow \textit{b}_{\textit{0}}, \quad \textit{c}\rightarrow 0, \quad as \quad {y} \rightarrow \infty , \end{aligned}$$

where $u_{e}$ is the external velocity and $\lambda$ is the stretching/shrinking parameter. $T _{w}$ and $T _{\infty }$ are the hybrid nanofluid temperature at surface and infinity respectively. The thermophysical properties of water, magnetite and copper nanoparticles are given in Table 1 while mathematical form to the thermophysical properties of nanofluid and hybrid nanofluid is given in Table 2. In Table 1, $\hbox {s}_{1}$ and $\hbox {s}_{2}$ are used respectively for the solid nanoparticles of $\hbox {Fe}_{3}\hbox {O}_{4}$ (magnetite) and Cu (Copper). f is used for the base fluid.

**Table 1:** Thermophysical properties of water and nanoparticles^3^.

Thermophysical properties	water	$\hbox {Fe}_{3}\hbox {O}_{4}$ (Magnetite)	Cu (Copper)
$\rho$ ($\hbox {kg/m}^{3}$)	$\rho _{f} = 997.1$	$\rho _{s_{1}} = 5200$	$\rho _{s_{2}} = 8933$
$\hbox {c} _{P}$ (J/kg K)	$(\hbox {c} _{P})_{f} = 4179$	$(\hbox {c} _{P})_{s_{1}} = 670$	$(\hbox {c} _{P})_{s_{2}} = 385$
*k* (W/m K)	$k_{f} = 0.613$	$k_{s_{1}}= 6$	$k_{s_{2}} = 401$
$\sigma \times 10^{-6}\,(\hbox {sm})^{-1}$	$\sigma _{f} = 0.05$	$\sigma _{s_{1}} = 0.025$	$\sigma _{s_{2}} = 59.6$
$\beta \times 10^{-5}\,(\hbox {K})^{-1}$	$\beta _{f} = 21$	$\beta _{s_{1}} = 1.3$	$\beta _{s_{2}} = 1.67$

**Table 2:** Formulations of thermophysical properties of nanofluid and hybrid nanofluid^27,33^.

Properties	Nanofluid ($\hbox {Fe}_{3}\hbox {O}_{4}/\hbox {H}_{2}\hbox {O}$)
Density ($\rho$)	$\rho _{nf} = (1 - \phi )\rho _{f}+ \phi \rho _{s}$
Heat capacity ($\rho \hbox {c} _{P}$)	($\rho \hbox {c} _{P}$)$_{nf}$ = (1 - $\phi$)($\rho \hbox {c} _{P}$)$_{f}$ + $\phi$($\rho \hbox {c} _{P}$)$_{s}$
Dynamic viscosity ($\mu$)	$\frac{\mu _{nf}}{\mu _{f}}= \frac{1}{(1 - \phi )^{2.5}}$
Thermal conductivity (*k*)	$\frac{k_{nf}}{k_{f}}$ = $\frac{k_{s} + 2k_{f} - 2\phi (k_{f} - k_{s})}{k_{s} + 2k_{f} + \phi (k_{f} - k_{s})}$
Electrical conductivity ($\sigma$)	$\frac{\sigma _{nf}}{\sigma _{f}}$ = 1 + $\frac{3(\sigma - 1)\phi }{(\sigma + 2) - (\sigma - 1)\phi }$, where $\sigma$ = $\frac{\sigma _{s}}{\sigma _{f}}$
Properties	Hybrid nanofluid (Cu-$\hbox {Fe}_{3}\hbox {O}_{4}$/H$_{2}$O)
Density ($\rho _{hnf}$)	$\rho _{hnf}$ = (1 - ($\phi _{1}$ + $\phi _{2}$))$\rho _{f}$ + $\phi _{1}\rho _{s_{1}}$ + $\phi _{2}\rho _{s_{2}}$
Heat capacity ($\rho$c$_{P}$)$_{hnf}$	($\rho$c$_{P}$)$_{hnf}$ = (1 - ($\phi _{1}$ + $\phi _{2}$))($\rho$c$_{P}$)$_{f}$ + $\phi _{1}$($\rho$c$_{P}$)$_{s_{1}}$ + $\phi _{2}$($\rho$c$_{P}$)$_{s_{2}}$
Dynamic viscosity ($\mu _{hnf}$)	$\frac{\mu _{hnf}}{\mu _{f}}$ = $\frac{1}{\left[1 - (\phi _{1} + \phi _{2})\right]^{2.5}}$
Thermal conductivity ($k_{_{hnf}}$)	$\frac{k_{hnf}}{k_{f}}$ = $\frac{\phi _{1}k_{s_{1}} + \phi _{2}k_{s_{2}} + 2\phi k_{f} + 2(\phi _{1}k_{s_{1}} + \phi _{2}k_{s_{2}}) - 2\phi ^{2}k_{f}}{\phi _{1}k_{s_{1}} + \phi _{2}k_{s_{2}} + 2\phi k_{f} - (\phi _{1}k_{s_{1}} + \phi _{2}k_{s_{2}}) + \phi ^{2}k_{f}}$
Electrical conductivity ($\sigma _{hnf}$)	$\frac{\sigma _{hnf}}{\sigma _{f}}$ = 1 + $\frac{3\left[\frac{\sigma _{1}\phi _{1} + \sigma _{2}\phi _{2}}{\sigma _{f}} - (\phi _{1} + \phi _{2})\right]}{2 + (\frac{\sigma _{1}\phi _{1} + \sigma _{2}\phi _{2}}{(\phi _{1} + \phi _{2})\sigma _{f}}) - \left[\frac{\sigma _{1}\phi _{1} + \sigma _{2}\phi _{2}}{\sigma _{f}} - (\phi _{1} + \phi _{2})\right]}$

Introducing the similarity transformations for (*f*, *g*), $\zeta$, $\theta$, $\varphi$ and $\varphi _{1}$ as the non-dimensional velocities, variable, temperature, homogeneous and heterogeneous chemical reactions respectively in the following form

9 $$\begin{aligned}{}&\psi ({x}, {y}, {t}) = {U_{\infty }}\sqrt{\nu _{f}{t}\cos \gamma + (\nu _{f}{x}/{U_{\infty }})\sin \gamma } {f}(\zeta ),\,\,{w} = {U_{\infty }}\sqrt{\nu _{f}{t}\cos \gamma + (\nu _{f}{x}/{U_{\infty }})\sin \gamma } {g}(\zeta )\nonumber \\&{u} = \frac{\partial \psi }{\partial y},\,{v} = - \frac{\partial \psi }{\partial x},\,\zeta = {y}/\sqrt{\nu _{f}{t}\cos \gamma + (\nu _{f}{x}/{U_{\infty }})\sin \gamma },\,\theta (\zeta ) = \frac{T -T_{\infty }}{T_{w} -T_{\infty }},\,{b} = {b_{0}}\varphi ,\,{c} = {b_{0}}\varphi _{1}, \end{aligned}$$

where $\psi$ is the stream function. $\nu _{f}$ is the kinematic viscosity. $\gamma$ is the leading edge accretion or ablation parameter and ($\nu _{f}{} \textit{t}\cos \gamma + (\nu _{f}{} \textit{x}/{U_{\infty }})\sin \gamma$) must be greater than zero^2–4^.

The continuity Eq. (1) is identically satisfied through Eq. (9). The different values are used from Tables 1, 2 & Eq. (9) to get the following equations from Eqs. (2–8) on the case when $D_{B} = D_{C}$^1^,

10 $$\begin{aligned}{}&\frac{1}{\Phi _{1}\Phi _{2}}{f^{\prime \prime \prime }} + \frac{1}{2}(\cos \gamma )\zeta {f}^{\prime \prime } + \frac{1}{2}(\sin \gamma ){f}{f}^{\prime \prime } - \Phi _{5}\dfrac{M((1 + m_{1}m_{2})f^{\prime } + m_{1}g)}{(1 + m_{1}m_{2})^{2} + m_{2}^{2}} + Gr\theta = 0, \end{aligned}$$

11 $$\begin{aligned}{}&\frac{1}{\Phi _{1}\Phi _{2}}{g^{\prime \prime }} + \frac{1}{2}(\sin \gamma ){f}{g}^{\prime } + \frac{1}{2}(\cos \gamma )\zeta {g}^{\prime \prime } + \Phi _{5}\dfrac{M(m_{1}f^{\prime } - (1 + m_{1}m_{2})g)}{(1 + m_{1}m_{2})^{2} + m_{2}^{2}} = 0, \end{aligned}$$

12 $$\begin{aligned}{}&\frac{1}{Pr}\frac{\Phi _{4}}{\Phi _{3}}\theta ^{\prime \prime } + \frac{1}{2}\left[(\sin \gamma )\textit{f} + (\cos \gamma )\zeta \right]\theta ^{\prime } + \frac{1}{\Phi _{1}\Phi _{3}}{} \textit{Ec}{f^{\prime \prime }}^{2} = 0, \end{aligned}$$

13 $$\begin{aligned}{}&\varphi ^{\prime \prime } + \frac{1}{2}{} \textit{Sc}\left[(\sin \gamma )\textit{f} + \zeta (\cos \gamma )\right]\varphi ^{\prime } - k_{2}\varphi (1 -\varphi )^{2} = 0, \end{aligned}$$

14 $$\begin{aligned}{}&{f} = 0, \, {f^{\prime }} = \lambda ,\, {g} = 0, \, \theta = 1,\, \varphi ^{\prime } = \textit{k}_{\textit{2}}\varphi \, at \, \zeta = 0, \end{aligned}$$

15 $$\begin{aligned}{}&{f^{\prime }} = 1,\,{g} = 0,\, \theta = 0,\,\varphi = 1\, at \, \zeta = \infty , \end{aligned}$$

where $\textit{Gr} = \left[\frac{g_{1}\beta _{T}(T_{w} - T_{\infty })}{\nu _{f}U_{\infty }}\right]\left[{} \textit{t}\cos \gamma + (\nu _{f}{} \textit{x}/{U_{\infty }})\sin \gamma )\right]$ is the thermal Grashof number, $\Phi _{1} = \left[1 - (\phi _{1} + \phi _{2})\right]^{2.5}$, $\Phi _{2}$ = $\phi _{1}\frac{\rho _{s_{1}}}{\rho _{f}} + \phi _{2}\frac{\rho _{s_{2}}}{\rho _{f}} + (1 - (\phi _{1} + \phi _{2}))$, $\Phi _{3}$ = $\phi _{1}\frac{(\rho c_{P})_{s_{1}}}{(\rho c_{P})_{f}} + \phi _{2}\frac{(\rho c_{P})_{s_{2}}}{(\rho c_{P})_{f}} + (1 - (\phi _{1} + \phi _{2}))$, $\Phi _{4} = \frac{\phi _{1}k_{s_{1}} + \phi _{2}k_{s_{2}} + 2\phi k_{f} + 2\phi (\phi _{1}k_{s_{1}} + \phi _{2}k_{s_{2}}) - 2\phi ^{2}k_{f}}{\phi _{1}k_{s_{1}} + \phi _{2}k_{s_{2}} + 2\phi k_{f} - \phi (\phi _{1}k_{s_{1}} + \phi _{2}k_{s_{2}}) + \phi ^{2}k_{f}}$, $\Phi _{5} = 1 + \frac{3\left[\frac{\sigma _{1}\phi _{1} + \sigma _{2}\phi _{2}}{\sigma _{f}} - (\phi _{1} + \phi _{2})\right]}{2 + \left[\frac{\sigma _{1}\phi _{1} + \sigma _{2}\phi _{2}}{(\phi _{1} + \phi _{2})\sigma _{f}}\right] - \left[\frac{\sigma _{1}\phi _{1} + \sigma _{2}\phi _{2}}{\sigma _{f}} - (\phi _{1} + \phi _{2})\right]}$. ($^{\prime }$) represents the derivative with respect to $\zeta$. ${M} = B^{2}_{0}\left[\nu _{hnf}{} \textit{t}\cos \gamma + (\nu _{hnf}{} \textit{x}/U_{\infty })\sin \gamma \right]$ is the magnetic field parameter, ${Pr} = \frac{\mu _{f}(c_{P})_{f}}{k_{f}}$ is the Prandtl number, $\textit{Ec} = \frac{(U_{\infty })^{2}}{(c_{P})_{f}(T_{w} - T_{\infty })}$ is the Eckert number, ${Sc} = \frac{\mu _{f}}{\rho _{f}D_{m}}$ is the Schmidt number, and $k_{1} = \frac{k_{c}b^{2}_{0}}{U}$^1^ is the strength of homogeneous chemical reaction and $k_{2} = \frac{k_{s}}{D_{B}}$ is the strength of heterogeneous chemical reaction.

It is important to investigate the physical quantities like local skin friction coefficients (Cfx,Cgz ) and Nusselt number Nux having extensive applications in industries as

16 $$\begin{aligned} {C_{f_{x}}} = \frac{\tau _{w_{x}}}{\rho _{hnf} U^{2}_{\infty }},\,{C_{g_{z}}} = \frac{\tau _{w_{z}}}{\rho U^{2}_{\infty }},\,\,{Nu} = \frac{q_{w}{x}}{{k_{hnf}}(-{T}_{\infty } + {T}_{w})}, \end{aligned}$$

where

17 $$\begin{aligned} \tau _{w_{x}} = \left[ \mu _{hnf}\frac{\partial u}{\partial y} \right]_{y=0},\,\tau _{w_{z}} = \left[ \mu _{hnf}\frac{\partial w}{\partial y} \right]_{y=0},\,{q_{w}} = -{k_{hnf}}\left[ \frac{\partial T}{\partial y} \right]_{y=0}. \end{aligned}$$

Note that $\tau _{w_{x}}$, $\tau _{w_{z}}$ and $q_{w}$ are known as wall frictions and heat transfer on the surface respectively.

Application of Eqs. (17) in (16) with Eq. (9), provide

18 $$\begin{aligned}{}&{C_{f_{x}}} = {f^{\prime \prime }}(0)({Re_{x}})^{\frac{-1}{2}}\frac{1}{\Phi _{1}}\frac{1}{\sqrt{\sin \gamma + \cos \gamma \sigma _{1}}},\,{C_{g_{z}}} = {g^{\prime }}(0)({Re_{x}})^{\frac{-1}{2}}\frac{1}{\Phi _{1}}\frac{1}{\sqrt{\sin \gamma + \cos \gamma \sigma _{1}}},\nonumber \\&{Nu_{x}} = - \theta ^{\prime }(0)({Re_{x}})^{\frac{1}{2}}\frac{1}{\sqrt{\sin \gamma + \cos \gamma \sigma _{1}}}, \end{aligned}$$

where $Re_{x} = \frac{Ux}{\nu _{hnf}}$ is the local Reynolds number with $\sigma _{1} = \frac{U_{\infty }t}{x}$ is the non-dimensional timing parameter.

## Entropy generation

The equation representing the entropy generation^34–40^ is

19 $$\begin{aligned} E^{\prime \prime \prime }_{gen} = \frac{k_{hnf}}{T^{2}_{\infty }}\left[\left(\frac{\partial T}{\partial x}\right)^{2} + \left(\frac{\partial T}{\partial y}\right)^{2}\right] + \frac{\mu _{hnf}}{T_{\infty }}\left[\frac{\partial u}{\partial y}\right]^{2} + \frac{RD}{b}\left[ \left( \frac{\partial b}{ \partial x}\right) ^{2}+\left( \frac{\partial b}{\partial y}\right) ^{2} \right] + \frac{RD}{T_{\infty }}\left[ \frac{\partial b}{\partial x}\frac{\partial T}{ \partial x}+\frac{\partial b}{\partial y}\frac{\partial T}{\partial y}\right] , \end{aligned}$$

where *R* is the ideal gas constant while *D* is the diffusivity. In Eq. (19), the first and second terms are respectively entropy generation due to heat transfer and viscous dissipation. The third and fourth terms are the diffusivity effects due to nanoparticles.

The characteristic entropy generation is

20 $$\begin{aligned} E^{\prime \prime \prime }_{0} = \frac{k_{hnf}(T_{w} - T_{\infty })^{2}}{x^{2}T^{2}_{\infty }}. \end{aligned}$$

The non-dimensional entropy generation rate $N_{G}(\zeta )$ is obtained with the help of Eq. (9) as

21 $$\begin{aligned} {N_{G}}(\zeta ) = \frac{E^{\prime \prime \prime }_{gen}}{E^{\prime \prime \prime }_{0}}. \end{aligned}$$

So

22 $$\begin{aligned} {N_{G}}(\zeta ) = \frac{5}{4}{} \textit{Re}(\theta ^{\prime })^{2} + \frac{5ReBr}{4(\theta _{w})^{2}}({f^{\prime \prime }})^{2} + \textit{Re}\frac{5}{4\varphi }\gamma _{1}\left[\frac{\varphi ^{\prime }}{\theta _{w}}\right]^{2} + \textit{Re}\frac{5}{4\Phi _{4}}\gamma _{1}\left[\frac{1}{\theta _{w}}\right]\theta ^{\prime }\varphi ^{\prime }, \end{aligned}$$

where $\textit{Re} = \frac{x^{2}}{\nu _{hnf}t\cos \gamma + (\nu _{hnf}x/U_{\infty })\sin \gamma }$ is the Reynolds number, $\theta _{w} = \frac{T_{w} - T_{\infty }}{T_{\infty }}$ is the temperature difference parameter, $\textit{Br} = \frac{\mu _{hnf} U^{2}_{\infty }}{k_{hnf} T_{\infty }}$ is the Brinkman number, $\gamma _{1} = \frac{RDb_{0}}{k_{hnf}}$ is the diffusion constant parameter due to nanoparticles concentration.

## Solution of the problem

Choosing the initial approximations and linear operators as

23 $$\begin{aligned}{}&{f_{0}}(\zeta ) = -\lambda \zeta + \zeta - \zeta \exp (-\zeta ),\,{g_{0}}(\zeta ) = \exp (-\zeta ),\,\theta _{0}(\zeta ) = \exp (-\zeta ),\,\varphi _{0}(\zeta ) = \zeta - \exp (-\zeta ), \end{aligned}$$

24 $$\begin{aligned}{}&{{\varvec{L}}}_{{f}} = {f^{\prime \prime \prime }},\,{{\varvec{L}}}_{{g}} = {g^{\prime \prime }},\,{{\varvec{L}}}_{\theta } = \theta ^{\prime \prime },\, {{\varvec{L}}}_{\varphi } = \varphi ^{\prime \prime } \end{aligned}$$

where

25 $$\begin{aligned} {{\varvec{L}}}_{f}\left[{E_{1}} + {E_{2}}\zeta + {E_{3}}\zeta ^{2}\right] = 0, \,{{\varvec{L}}}_{g}\left[{E_{4}} + {E_{5}}\zeta \right] = 0,\, {{\varvec{L}}}_{\theta }\left[{E_{6}} + {E_{7}}\zeta \right] = 0,\,{{\varvec{L}}}_{\varphi }\left[{E_{8}} + {E_{9}}\zeta \right] = 0, \end{aligned}$$

and $E_{i}$(*i* = 1-9) are the arbitrary constants in general solution.

### Zeroth order deformation problems

26 $$\begin{aligned}{}&\aleph _{f}[f(\zeta , {j})] = \frac{1}{\Phi _{1}\Phi _{2}}\frac{\partial ^{3}f(\zeta , {j})}{\partial \zeta ^{3}} + \frac{1}{2}(\sin \gamma ) f(\zeta , {j}) \frac{\partial ^{2}f(\zeta , j)}{\partial \zeta ^{2}} + \frac{1}{2}(\cos \gamma )\zeta \frac{\partial ^{2}f(\zeta , j)}{\partial \zeta ^{2}} \nonumber \\&\quad -\Phi _{5}\dfrac{M}{(1 + m_{1}m_{2})^{2} + m_{2}^{2}}\left[\frac{(1 + m_{1}m_{2})\partial f(\zeta , {j})}{\partial \zeta } + m_{1}g(\zeta , {j})\right] + Gr\theta (\zeta , j), \end{aligned}$$

27 $$\begin{aligned}{}&\aleph _{g}[g(\zeta , {j})] = \frac{1}{\Phi _{1}\Phi _{2}}\frac{\partial ^{2}g(\zeta , {j})}{\partial \zeta ^{2}} + \frac{1}{2}(\sin \gamma ) f(\zeta , {j}) \frac{\partial g(\zeta , j)}{\partial \zeta } + \frac{1}{2}(\cos \gamma )\zeta \frac{\partial ^{2}g(\zeta , j)}{\partial \zeta ^{2}} \nonumber \\&\quad +\Phi _{5}\dfrac{M}{(1 + m_{1}m_{2})^{2} + m_{2}^{2}}\left[\frac{m_{1}\partial f(\zeta , {j})}{\partial \zeta } - (1 + m_{1}m_{2})g(\zeta , {j})\right], \end{aligned}$$

28 $$\begin{aligned}{}&\aleph _{\theta }[f(\zeta , j), \theta (\zeta , j)] = \frac{1}{{Pr}}\frac{\Phi _{4}}{\Phi _{3}}\frac{\partial ^{2} \theta (\zeta , j)}{\partial \zeta ^{2}} + \frac{1}{2}\left[(\cos \gamma )\zeta + (\sin \gamma )f(\zeta , {j})\right]\frac{\partial \theta (\zeta , j)}{\partial \zeta } + \frac{1}{\Phi _{1}\Phi _{3}}{} \textit{Ec}\left[\frac{\partial ^{2} \textit{f}(\zeta , j)}{\partial \zeta ^{2}}\right]^{2}, \end{aligned}$$

29 $$\begin{aligned}{}&\aleph _{\varphi }[f(\zeta , j), \varphi (\zeta , j)] = \frac{\partial ^{2} \varphi (\zeta , j)}{\partial \zeta ^{2}} + \frac{1}{2}{} \textit{Sc}\left[(\cos \gamma )\zeta + (\sin \gamma )f(\zeta , {j})\right]\frac{\partial \varphi (\zeta , j)}{\partial \zeta } - k_{2}\varphi (\zeta , j)\left[1 - \varphi (\zeta , j)\right]^{2}, \end{aligned}$$

where $\aleph$ is the nonlinear operator and *j* is the embedding parameter in such a way that *j* $\in$ [0, 1].

Considering the deformations with boundary conditions and $\hslash _{f}$, $\hslash _{g}$, $\hslash _{\theta }$ and $\hslash _{\varphi }$ as the auxiliary non-zero parameters

30 $$\begin{aligned}{}&(1 - {j}) {{\varvec{L}} _{{\varvec{f}} }} [{f}(\zeta , {j}) - {f_{0}}(\zeta )] = {j} \hslash _{f} \aleph _{f} [{f}(\zeta , {j})], \end{aligned}$$

31 $$\begin{aligned}{}&(1 - {j}){{\varvec{L}} _{{\varvec{g}} }} [{g}(\zeta , {j}) - {g_{0}}(\zeta )] = {j}\hslash _{g}\aleph _{g} [{g}(\zeta , {j})], \end{aligned}$$

32 $$\begin{aligned}{}&(1 - {j}) {{\varvec{L}} _{\theta } } [\theta (\zeta , {j}) - \theta _{0}(\zeta )] = {j} \hslash _{\theta } \aleph _{\theta } [{f}(\zeta , {j}), \theta (\zeta , {j})], \end{aligned}$$

33 $$\begin{aligned}{}&(1 - {j}) {{\varvec{L}} _{\varphi } } [\varphi (\zeta , {j}) - \varphi _{0}(\zeta )] = {j} \hslash _{\varphi } \aleph _{\varphi } [{f}(\zeta , {j}), \varphi (\zeta , {j})], \end{aligned}$$

34 $$\begin{aligned}{}&{f} (0, j) = 0,\, {f^{\prime }} (0, j) = \lambda ,\, {f^{\prime }} (\infty , j) = 1, \end{aligned}$$

35 $$\begin{aligned}{}&g (0, j) = 0,\, g(\infty , j) = 0, \end{aligned}$$

36 $$\begin{aligned}{}&\theta (0, j) = 1,\, \theta (\infty , j) = 0, \end{aligned}$$

37 $$\begin{aligned}{}&\varphi ^{\prime } (0, j) = k_{2}\varphi (0, j),\, \varphi (\infty , j) = 1. \end{aligned}$$

For *j* = 0 and *j* = 1, the equations (30-33) provide

38 $$\begin{aligned}{}&{j} = 0 \Rightarrow {f} (\zeta , 0) = f_{0}(\zeta ) \,\, and \,\, {j} = 1 \Rightarrow {f}(\zeta , 1) = f(\zeta ), \end{aligned}$$

39 $$\begin{aligned}{}&{j} = 0 \Rightarrow {g} (\zeta , 0) = g_{0}(\zeta ) \,\, and \,\, {j} = 1 \Rightarrow {g}(\zeta , 1) = g(\zeta ), \end{aligned}$$

40 $$\begin{aligned}{}&{j} = 0 \Rightarrow \theta (\zeta , 0) = \theta _{0}(\zeta ) \,\, and \,\, {j} = 1 \Rightarrow \theta (\zeta , 1) = \theta (\zeta ), \end{aligned}$$

41 $$\begin{aligned}{}&{j} = 0 \Rightarrow \varphi (\zeta , 0) = \varphi _{0}(\zeta ) \,\, and \,\, {j} = 1 \Rightarrow \varphi (\zeta , 1) = \varphi (\zeta ), \end{aligned}$$

Applying the Taylor series to Eqs. (38–41), it is obtained

42 $$\begin{aligned}{}&{f}(\zeta , {j}) = {f_{0}}(\zeta ) + \sum ^{\infty }_{m = 1} {f_{m}}(\zeta ){j^{m}}, \,\, {f_{m}}(\zeta ) = \frac{1}{m!} \frac{\partial ^{m} {f}(\zeta , {j})}{\partial j^{m}}\mid _{j=0}, \end{aligned}$$

43 $$\begin{aligned}{}&{g}(\zeta , {j}) = {g_{0}}(\zeta ) + \sum ^{\infty }_{m = 1} {g_{m}}(\zeta ){j^{m}}, \,\, {g_{m}}(\zeta ) = \frac{1}{m!} \frac{\partial ^{m} {g}(\zeta , {j})}{\partial j^{m}}\mid _{j=0}, \end{aligned}$$

44 $$\begin{aligned}{}&\theta (\zeta , {j}) = \theta _{0}(\zeta ) + \sum ^{\infty }_{m = 1} \theta _{m}(\zeta ){j^{m}}, \,\, \theta _{m}(\zeta ) = \frac{1}{m!} \frac{\partial ^{m} \theta (\zeta , {j})}{\partial j^{m}}\mid _{j=0}, \end{aligned}$$

45 $$\begin{aligned}{}&\varphi (\zeta , {j}) = \varphi _{0}(\zeta ) + \sum ^{\infty }_{m = 1} \varphi _{m}(\zeta ){j^{m}}, \,\, \varphi _{m}(\zeta ) = \frac{1}{m!} \frac{\partial ^{m} \varphi (\zeta , {j})}{\partial j^{m}}\mid _{j=0}. \end{aligned}$$

The convergence is achieved on the suitable values of $\hslash _{f}$, $\hslash _{g}$, $\hslash _{\theta }$, and $\hslash _{\varphi }$ in Eqs. (42–45) at *j* = 1. So Eqs. (42–45) switch to

46 $$\begin{aligned} {f}(\zeta )&= {f_{0}}(\zeta ) + \sum ^{\infty }_{m = 1}{f_{m}}(\zeta ) , \end{aligned}$$

47 $$\begin{aligned} {g}(\zeta )&= {g_{0}}(\zeta ) + \sum ^{\infty }_{m = 1}{g_{m}}(\zeta ) , \end{aligned}$$

48 $$\begin{aligned} \theta (\zeta )&= \theta _{0}(\zeta ) + \sum ^{\infty }_{m = 1}\theta _{m}(\zeta ), \end{aligned}$$

49 $$\begin{aligned} \varphi (\zeta )&= \varphi _{0}(\zeta ) + \sum ^{\infty }_{m = 1}\varphi _{m}(\zeta ). \end{aligned}$$

### mth order deformation problems

Deformation for the mth order of the Eqs. (30) and (34) is

50 $$\begin{aligned}{}&{{\varvec{L}}_{{\varvec{f}}}}[{f_{m}}(\zeta ) - \chi _{m}{f_{m-1}}(\zeta )] = \hslash _{f} \mathfrak {R}^{f}_{m}(\zeta ), \end{aligned}$$

51 $$\begin{aligned}{}&f_{m}(0) = 0,\,\, f^{\prime }_{m}(0) = 0,\,\, f^{\prime }_{m}(\infty ) = 0, \end{aligned}$$

where

52 $$\begin{aligned}{}&\mathfrak {R}_{m}^{f}(\zeta ) = \frac{1}{\Phi _{1}\Phi _{2}}f^{\prime \prime \prime }_{m-1} + \frac{1}{2}(\sin \gamma )\sum ^{m - 1}_{k = o}\left[ f_{m - 1 - k} f^{\prime \prime }_{k} \right] + \frac{1}{2}(\cos \gamma )\zeta f^{\prime \prime }_{m - 1} \nonumber \\&\quad -\Phi _{5}\dfrac{M}{(1 + m_{1}m_{2})^{2} + m_{2}^{2}}\left[(1 + m_{1}m_{2})f^{\prime }_{m - 1} + {m_{1}}{} g _{m-1}\right] + Gr\theta _{m-1}. \end{aligned}$$

mth order deformation for Eqs. (31) and (35) is

53 $$\begin{aligned} {{\varvec{L}}_{{\varvec{g}}}}[{g_{m}}(\zeta ) - \chi _{m}{g_{m-1}}(\zeta )] = \hslash _{g} \mathfrak {R}^{g}_{m}(\zeta ), \end{aligned}$$

54 $$\begin{aligned}{}&g_{m}(0) = 0,\,\,g_{m}(\infty ) = 0, \end{aligned}$$

where

55 $$\begin{aligned}{}&\mathfrak {R}_{m}^{g}(\zeta ) = \frac{1}{\Phi _{1}\Phi _{2}}g^{\prime \prime }_{m-1} + \frac{1}{2}(\sin \gamma )\sum ^{m - 1}_{k = o}\left[ f_{m - 1 - k} g^{\prime }_{k} \right] + \frac{1}{2}(\cos \gamma )\zeta g^{\prime \prime }_{m - 1} \nonumber \\&\quad +\Phi _{5}\dfrac{M}{(1 + m_{1}m_{2})^{2} + m_{2}^{2}}\left[ m_{1} f^{\prime }_{m - 1} - (1 + m_{1}m_{2})g _{m-1} \right]. \end{aligned}$$

mth order deformation for Eqs. (32) and (36) is

56 $$\begin{aligned}{}&{{\varvec{L}}_{\theta }}[\theta _{m}(\zeta ) - \chi _{m}\theta _{m-1}(\zeta )] = \hslash _{\theta } \mathfrak {R}^{\theta }_{m}(\zeta ), \end{aligned}$$

57 $$\begin{aligned}{}&\theta _{m}(0) = 0,\,\, \theta _{m}(\infty ) = 0, \end{aligned}$$

where

58 $$\begin{aligned} \mathfrak {R}_{m}^{\theta }(\zeta ) = \frac{1}{Pr }\frac{\Phi _{4}}{\Phi _{3}}\theta ^{\prime \prime }_{m-1} + \frac{1}{2}(\cos \gamma )\zeta \theta ^{\prime }_{m-1} + \frac{1}{2}\sin \gamma \sum ^{m - 1}_{k = o} f_{m - 1 - k}\theta ^{\prime }_{k} + \frac{1}{\Phi _{1}\Phi _{3}} \textit{Ec}\sum ^{m - 1}_{k = o}f^{\prime \prime }_{m - 1 - k}f^{\prime \prime }_{k}. \end{aligned}$$

mth order deformation for Eqs. (33) and (37) is

59 $$\begin{aligned} {{\varvec{L}}_{\varphi }}[\varphi _{m}(\zeta ) - \chi _{m}\varphi _{m-1}(\zeta )] = \hslash _{\varphi } \mathfrak {R}^{\varphi }_{m}(\zeta ), \end{aligned}$$

60 $$\begin{aligned}{}&\varphi ^{\prime } (0) = k_{2}\varphi (0),\, \varphi (\infty ) = 0, \end{aligned}$$

where

61 $$\begin{aligned}{}&\mathfrak {R}^{\varphi }_{m}(\zeta ) = \varphi ^{\prime \prime }_{m-1} + \frac{1}{2}{} \textit{Sc}(\cos \gamma )\zeta \varphi ^{\prime }_{m-1} + \frac{1}{2}{} \textit{Sc}\sin \gamma \sum ^{m - 1}_{k = o} f_{m - 1 - k}\varphi ^{\prime }_{k} - k_{2}\varphi _{m-1} - k_{2}\sum ^{m - 1}_{k = o} \varphi _{m - 1 - k} \sum ^{k}_{l = o} \left[ \varphi _{k - l} \varphi _{l}\right]\nonumber \\&\quad + 2 k_{2}\sum ^{m - 1}_{k = o} \varphi _{m - 1 - k}\varphi _{k}. \end{aligned}$$

62 $$\begin{aligned}{}&\chi _{m} = \left\{ \begin{array}{ll} 0, &{} {{m} \leqslant 1} \\ 1, &{} {{m} > 1.} \end{array} \right. \end{aligned}$$

Using the particular solutions $f^{*}_{m}$($\zeta$), $g^{*}_{m}$($\zeta$), $\theta ^{*}_{m}$($\zeta$) and $\varphi ^{*}_{m}$($\zeta$), the general solutions of Eqs. (50), (53), (56) and (59) are represented as

63 $$\begin{aligned}{}&{f_{m}}(\zeta ) = {f^{*}_{m}}(\zeta ) + {E_{1}} + {E_{2}}\zeta + {E_{3}}\zeta ^{2}, \end{aligned}$$

64 $$\begin{aligned}{}&g_{m}(\zeta ) = g^{*}_{m}(\zeta ) + {E_{4}} + {E_{5}}\zeta , \end{aligned}$$

65 $$\begin{aligned}{}&\theta _{m}(\zeta ) = \theta ^{*}_{m}(\zeta ) + {E_{6}} + {E_{7}}\zeta , \end{aligned}$$

66 $$\begin{aligned}{}&\varphi _{m}(\zeta ) = \varphi ^{*}_{m}(\zeta ) + {E_{8}} + {E_{9}}\zeta . \end{aligned}$$

### Comparison of the present work

Relevant study^3^ is followed to compare the results. Using HAM solution, the results are computed for $f^{\prime \prime }$(0) in the consideration of leading edge accretion rate and leading edge accretion or ablation parameter $\gamma$ for the magnetite nanoparticles. The present results show a nice agreement with the results of^3^.

**Table 3:** Comparison of $f^{\prime \prime }$(0) values for accretion rate and the parameter $\gamma$.

Accretion rate	$\gamma$	Ilias et al.^3^	Present work
Rayleigh-Stokes	0	6.81462 $\times$ 10$^{-1}$	6.78462 $\times$ 10$^{-1}$
(0.7596 $\times$ 10$^{+1}$)U$_{\infty }$	$\frac{\pi }{24}$	6.92736 $\times$ 10$^{-1}$	6.12336 $\times$ 10$^{-1}$
(0.3732 $\times$ 10$^{+1}$)U$_{\infty }$	$\frac{\pi }{12}$	6.97910 $\times$ 10$^{-1}$	6.56410 $\times$ 10$^{-1}$
(0.1732) $\times$ 10$^{+1}$U$_{\infty }$	$\frac{\pi }{6}$	6.90109 $\times$ 10$^{-1}$	6.88109 $\times$ 10$^{-1}$
U$_{\infty }$	$\frac{\pi }{4}$	6.57763 $\times$ 10$^{-1}$	6.56563 $\times$ 10$^{-1}$
(5.77 $\times$ 10$^{-1}$)U$_{\infty }$	$\frac{\pi }{3}$	5.99310 $\times$ 10$^{-1}$	5.10310 $\times$ 10$^{-1}$
(2.68 $\times$ 10$^{-1}$)U$_{\infty }$	$\frac{5\pi }{12}$	5.10452 $\times$ 10$^{-1}$	5.21452 $\times$ 10$^{-1}$
(1.32 $\times$ 10$^{-1}$)U$_{\infty }$	$\frac{11\pi }{24}$	4.51264 $\times$ 10$^{-1}$	4.44264 $\times$ 10$^{-1}$
Blasius	$\frac{\pi }{2}$	3.77490 $\times$ 10$^{-1}$	3.6490 $\times$ 10$^{-1}$

## Results and discussion

Analytical solution of the Eqs. (10–15) is obtained via homotopy analysis method (HAM)^41^ and then Eq. (22) is solved. All the numerical values of thermophysical properties are used from Tables 1 and 2 in computing the solution. For the influential role of parameters in flow, heat and mass transfer as well as entropy generation, *Gr* = 0.30, $m_{1}$ = 1.00, $m_{2}$ = 0.20, *M* = 1.00, $\lambda$ = 0.25, *Sc* = 0.10, $\gamma$ = $\frac{\pi }{3}$, *Pr* = 6.20, *Re* = 0.10, *Br* = 0.50, $\gamma _{1}$ = 0.40 and $\theta _{w}$ = 0.10, are used. Comparison is made with the existing literature^3^ which can be seen in Table 3. The physical sketch of the problem is shown in Fig. 1. The *h*-curves are given in Figs. 2, 3, 4 and 5. The parameters effects are depicted in graphs through Figs. 6, 7, 8, 9, 10, 11, 12, 13, 14, 15, 16, 17, 18, 19, 20, 21, 22, 23, 24, 25, 26, 27, 28, 29, 30, 31, 32, 33, 34, 35, 36, 37, 38 and 39.

Figure 6 demonstrates the effect of Hall current parameter $m_{1}$ on the velocity $f^{\prime }(\zeta )$. The axial flow decreases progressively as $m_{1}$ increases. This is due to the fact that for the higher values of $m_{1}$, the magnitude of $m_{1}$ in the denominator in $\dfrac{M((1 + m_{1}m_{2})f^{\prime } + m_{1}g)}{(1 + m_{1}m_{2})^{2} + m_{2}^{2}}$ is very high which exerts the strong resistive effect of Hall parameter $m_{1}$. Similar observations are received from Fig. 7 where velocity $f^{\prime }(\zeta )$ is shown to decreasing for ion-slip parameter $m_{2}$. The reason is that in $\dfrac{M((1 + m_{1}m_{2})f^{\prime } + m_{1}g)}{(1 + m_{1}m_{2})^{2} + m_{2}^{2}}$, the $m_{2}$ appears in squaring form which results in the deceleration of hybrid nanofluid flow of Cu-$\hbox {Fe}_{3}\hbox {O}_{4}$/water. Figure 8 reveals the effect of the magnetic field parameter *M* on axial velocity $f^{\prime }(\zeta )$. For the positive values of *M*, a clear reduction in the flow of Cu-$\hbox {Fe}_{3}\hbox {O}_{4}$/water is marked. The magnetohydrodynamic drag force $- \Phi _{5}\dfrac{M((1 + m_{1}m_{2})f^{\prime } + m_{1}g)}{(1 + m_{1}m_{2})^{2} + m_{2}^{2}}$ is strongly related to the magnetic field parameter *M*, enforcing that for *M* = 1.00, 10.00, 19.00, 28.00, the retardation is extremely high. The velocity components [$f^{\prime }(\zeta )$ & *g*($\zeta$)] provide the negative contribution, therefore the overall influence of surging *M* yield a high negative body force. The effect of thermal buoyancy parameter *Gr* on axial velocity $f^{\prime }(\zeta )$ is depicted in Fig. 9. Through convection, the nanoparticles are excited, consequently, the flow is enhanced. It should be noted that for *Gr* = 0, the present system corresponds to the pure diffusive boundary layer which has a complete absence of convection. Figure 10 demonstrates the role of leading edge accretion/ablation parameter $\gamma$. The flow of hybrid nanofluid (Cu-$\hbox {Fe}_{3}\hbox {O}_{4}$/water) is decreased. Different cases depend on the values of $\gamma$. The case, $0 < \gamma \le \frac{\pi }{2}$, corresponds to the leading edge accretion with the rate $U_{\infty } \cot \gamma$. The case $\frac{-\pi }{4} \le \gamma < 0$, refers to backward boundary layer with trailing edge accretion. The case $\gamma = 0$, is relevant to Rayleigh-Stokes problem and $\gamma$ = $\frac{\pi }{2}$, relates to the Blasius flat plate. Figure 11 depicts that velocity $f^{\prime }(\zeta )$ accelerates with stretching parameter $\lambda$. $\lambda$ > 0 shows the stretching phenomena, $\lambda$ < 0 corresponds to shrinking case and $\lambda$ = 0 is used for the static surface. Prandtl number *Pr* refers to the nanoparticles concentrations, therefore on increasing the Prandtl number, axial velocity $f^{\prime }(\zeta )$ is reduced as shown in Fig. 12. The reason is that due to the addition of nanoparticles, the thickness of the hybrid nanofluid and Brownian motion of nanoparticles are increased which resists the flow. Figure 13 is related to the Eckert number *Ec* and axial velocity $f^{\prime }(\zeta )$. Velocity is easily enhanced on viscous dissipative effect.

The important outcome of this study is shown in Figs. 14 and 15 where the transverse velocity ${g}(\zeta )$ tends to decreasing on increasing ($m_{1}$ & $m_{2}$). The reasons is that for the high values of ($m_{1}$ & $m_{2}$), the effect of magnetic field is prominent. Therefore, the resistive effect of magnetic field is quite active. Figure 16 shows that the transverse velocity is decreased with the greater values of magnetic field parameter *M*. The magnetohydrodynamic drag force $- \Phi _{5}\dfrac{M((1 + m_{1}m_{2})f^{\prime } + m_{1}g)}{(1 + m_{1}m_{2})^{2} + m_{2}^{2}}$ in Eq. (11) is responsible to reduce the transverse velocity ${g}(\zeta )$. The thermal buoyancy parameter namely Grashof number *Gr* is a convection parameter, so it increases the motion of nanoparticles through convection as shown in Fig. 17. Figure 18 reports that leading edge accretion/ablation parameter $\gamma$ reduces the transverse velocity $\textit{g}(\zeta )$ for $\gamma = \frac{\pi }{2}$, $\frac{\pi }{3}$, $\frac{\pi }{4}$, $\frac{\pi }{5}$ (decreasing values of angle $\gamma$). The stretching parameter $\lambda$ in Fig. 19 shows that dimensionless transverse velocity ${g}(\zeta )$ augments to be maximized. The effect is more pronounced and the boundary layer thickness is expanded.

Figures 20 and 21 show that heat transfer is decreased for the various increasing values of ($m_{1}$ & $m_{2}$). The thermal boundary layer thickness is reduced. Considering Fig. 22, it has been shown that temperature distribution $\theta$($\zeta$) is minimum. A decrease in thermal boundary layer happens and so there is no change in the heat transfer. The thermal Grashof number *Gr* indicates the existence of convection so *Gr* > 0, generates the heating system. Increasing of *Gr*, causes the surge in buoyancy force which increases the temperature through convection as demonstrated by Fig. 23. It has been noticed in Fig. 24, that the temperature $\theta$($\zeta$) is reduced at $\gamma$ = $\frac{\pi }{2}$, $\frac{\pi }{3}$, $\frac{\pi }{4}$, $\frac{\pi }{5}$. It is observed that the thermal boundary layer thickness is decreased. For $\gamma$ = 0$^{o}$, there is no increasing or decreasing effect in the temperature. Figure 25 specifies the effect of stretching parameter $\lambda$ on temperature $\theta$($\zeta$). For $\lambda$ > 0, the thickness of thermal boundary layer is low due to which heat transfer is minimum. Prandtl number influence on temperature $\theta$($\zeta$) is examined in Fig. 26. The energy of the nanoparticles is transferred to the environment which cause to decrease the temperature. It leads to low heat transfer and the thickness of thermal boundary layer. *Pr* $<<$ 1 signifies that the thermal diffusivity prevails while *Pr* $>>$ 1 corresponds to the momentum diffusivity dominates the behavior. These characteristics are different for different fluids. For example ranges are 0.70 $\le$ *Pr* $\le$ 1.00, 1.00 $\le$ *Pr* $\le$ 10.00, 0.001 $\le$ *Pr* $\le$ 0.03 and 50.00 $\le$ *Pr* $\le$ 2000.00 for gases, water, liquid metals and oils respectively. Figure 27 shows that the Eckert number *Ec* boosts the thermal boundary layer and hence heat transfer.

The concentration of homogeneous chemical reactions $\varphi (\zeta )$ and Hall parameter $m_{1}$ role are shown through Fig. 28. It is revealed that $\varphi (\zeta )$ tends to minimum on the different values of $m_{1}$. The reason is that due to strong magnetic field, the nanoparticles are restricted in a region and there is very less opportunity for chemical reaction. Figures 29 and 30 exhibits the boosting up characteristics of parameters $m_{2}$ & *M* respectively. Figure 31 is constructed to show the effect of thermal buoyancy parameter *Gr*. With the increment of *Gr*, the heat generation exists consequently, the hybrid nanofluid (Cu-$\hbox {Fe}_{3}\hbox {O}_{4}$/water) temperature augments through convection which causes an upgrade in the homogeneous chemical reaction $\varphi (\zeta )$. The rise in Cu-$\hbox {Fe}_{3}\hbox {O}_{4}$/water temperature has the susceptibility to rise the concentration of homogeneous chemical reaction $\varphi (\zeta )$. Figure 32 illustrates the performance of leading accretion/ablation parameter $\gamma$ and concentration of homogeneous chemical reaction $\varphi (\zeta )$. With the given values of $\gamma$, $\varphi (\zeta )$ is amplified. Stretching/shrinking parameter $\lambda$ and $\varphi (\zeta )$ are depicted in Fig. 33. There is a very high change in the concentration. Figure 34 elucidates that the Schmidt number *Sc* proceeds to enhance the species concentration. The effect of strength of heterogeneous chemical reaction parameter $k_{2}$ and concentration of homogeneous chemical reaction $\varphi (\zeta )$ are shown in Fig. 35. It is depicted that the different values of $k_{2}$ decline the $\varphi (\zeta )$.

Entropy generation rate $N_{G}$($\zeta$) and Reynolds number *Re* are projected in Fig. 36. Due to increasing values of *Re*, viscosity of the nanoparticles increases which generate more resistive force to the flow and hence enhances the kinetic energy. It is reported that the irreversibility of the system increases with the Reynolds number *Re* which complement figure 4 of study^18^ with respect to increasing behavior. Similar increasing behavior of entropy generation rate $N_{G}$($\zeta$) has been shown for the Brinkman number *Br* in Fig. 37. The temperature difference parameter $\theta _{w}$ has the same increasing behavior for entropy generation rate $N_{G}$($\zeta$) in Fig. 38 which is already reported in figure 5 of study^18^. The progress of diffusion constant parameter $\gamma _{1}$ due to nanoparticle concentration of hybrid nanofluid Cu-$\hbox {Fe}_{3}\hbox {O}_{4}$/water is represented in Fig. 39 which has the same increasing behavior like the entropy behavior in Figs. 4 and 5 of the study^18^.

**Figure 2:**
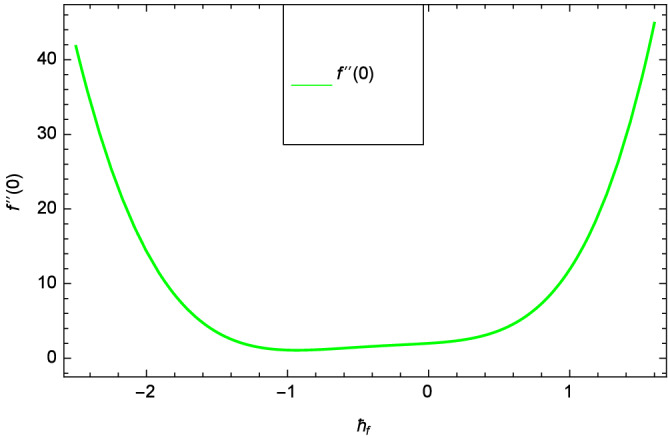
$$h_{f}$$ curve of *f*($$\zeta$$).

**Figure 3:**
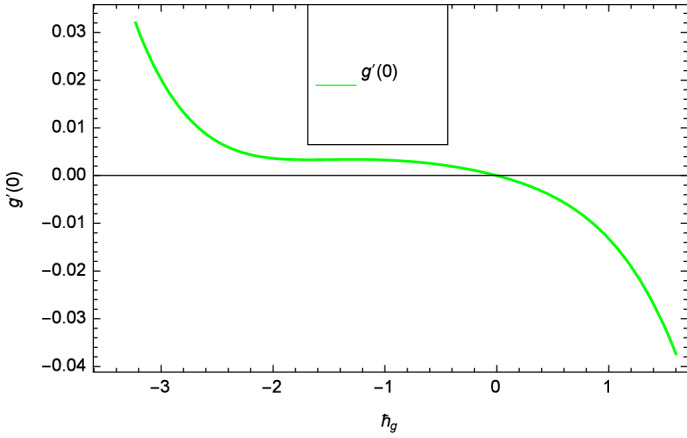
$$h_{g}$$ curve of *g*($$\zeta$$).

**Figure 4:**
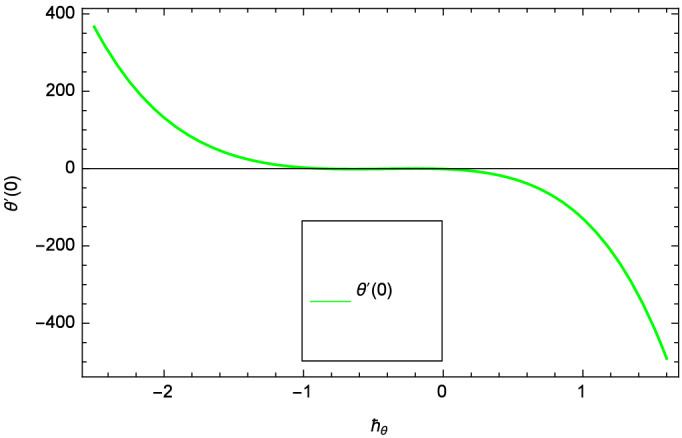
$$h_{\theta }$$ curve of $$\theta$$($$\zeta$$).

**Figure 5:**
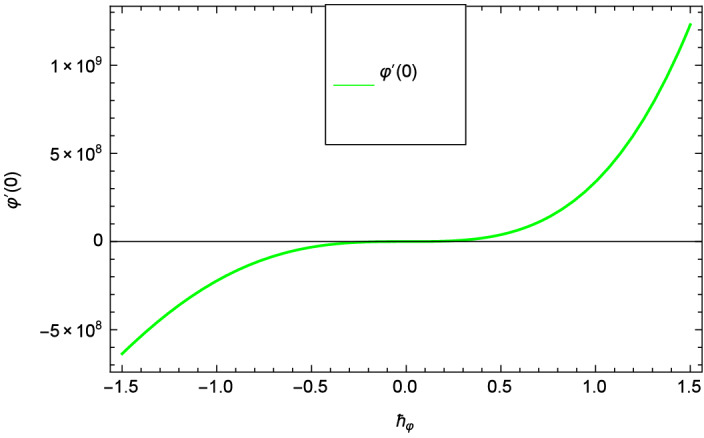
$$h_{\varphi }$$ curve of $$\varphi$$($$\zeta$$).

**Figure 6:**
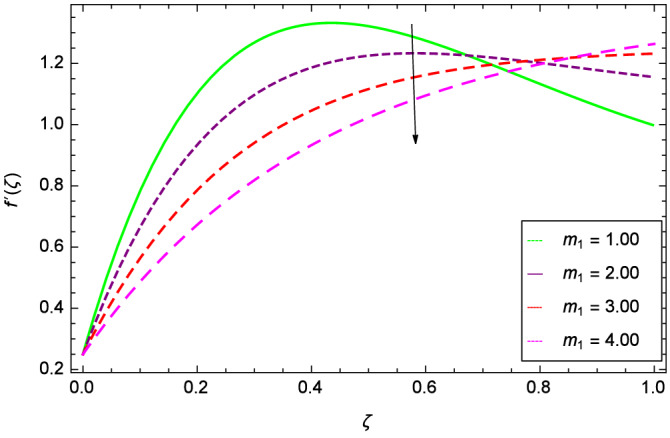
Parameter effect on velocity $f^{\prime }(\zeta )$ profile.

**Figure 7:**
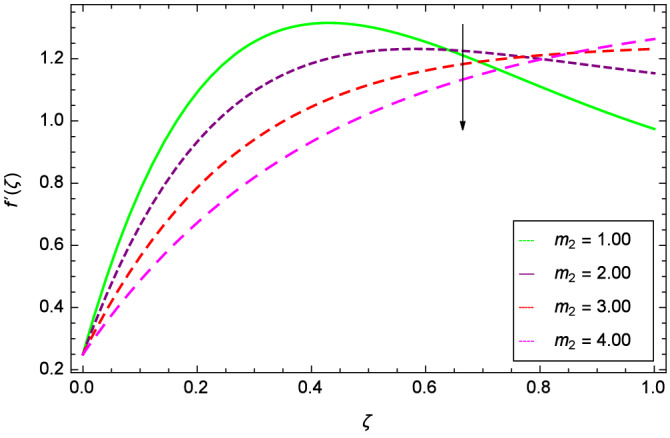
Parameter effect on velocity $f^{\prime }(\zeta )$ profile.

**Figure 8:**
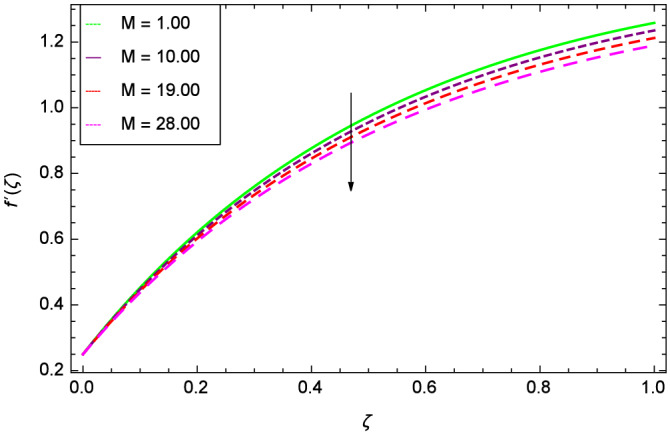
Parameter effect on velocity $f^{\prime }(\zeta )$ profile.

**Figure 9:**
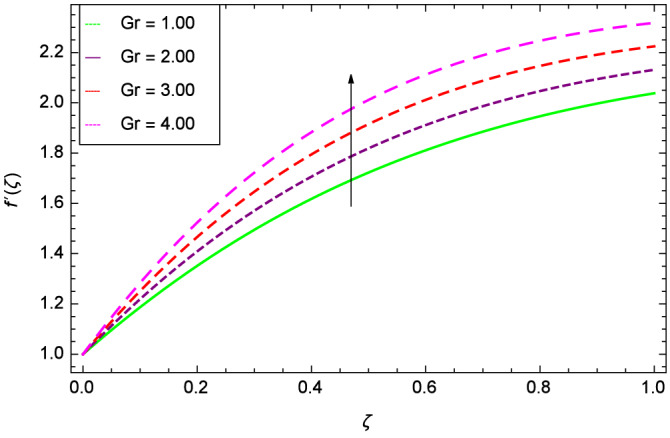
Parameter effect on velocity $f^{\prime }(\zeta )$ profile.

**Figure 10:**
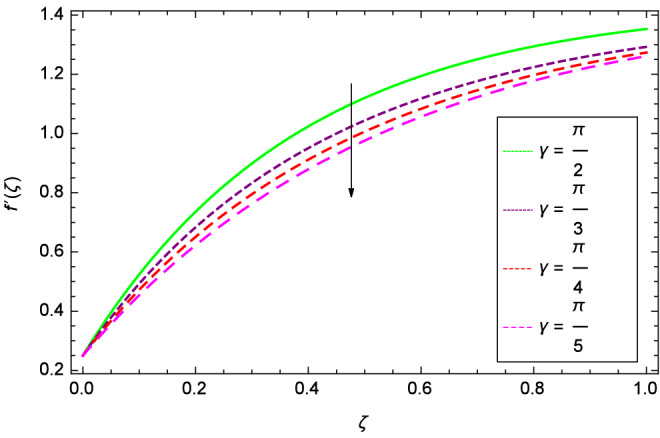
Parameter effect on velocity $f^{\prime }(\zeta )$ profile.

**Figure 11:**
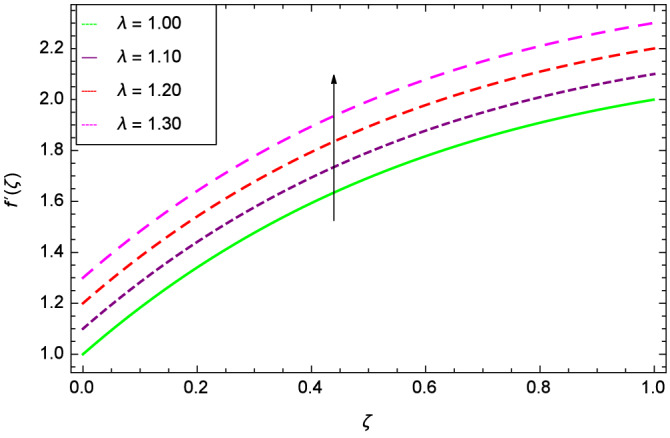
Parameter effect on velocity $f^{\prime }(\zeta )$ profile.

**Figure 12:**
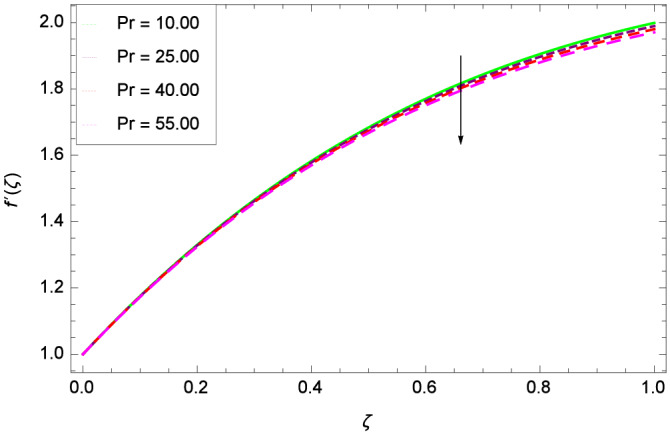
Parameter effect on velocity $f^{\prime }(\zeta )$ profile.

**Figure 13:**
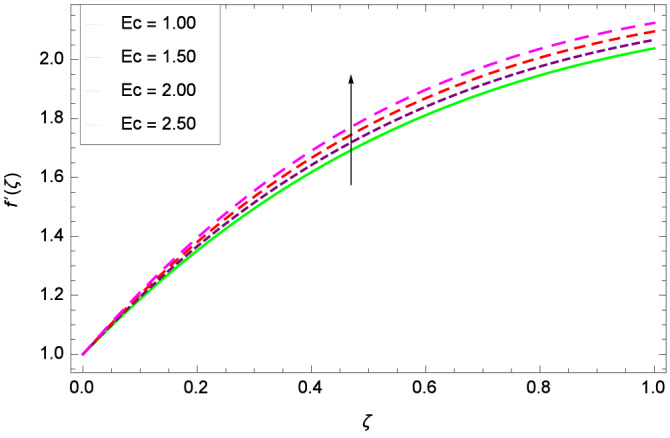
Parameter effect on velocity $f^{\prime }(\zeta )$ profile.

**Figure 14:**
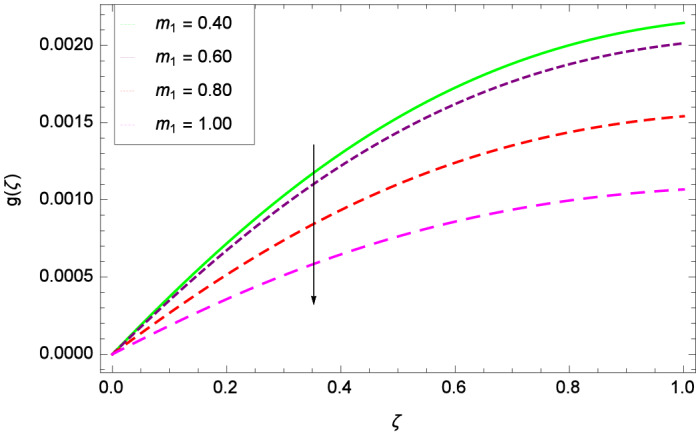
Parameter effect on velocity *g*($\zeta$) profile.

**Figure 15:**
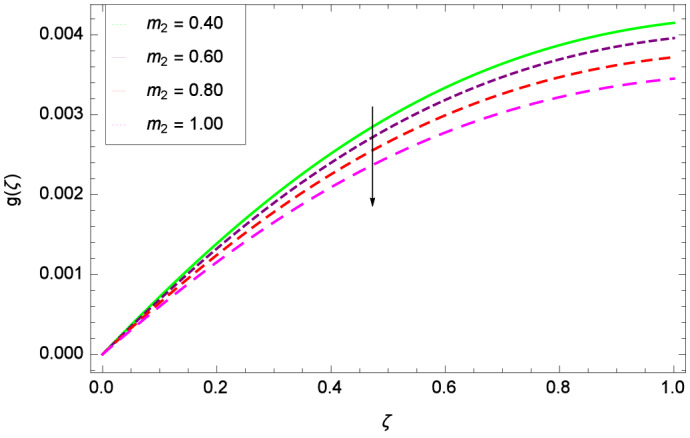
Parameter effect on velocity *g*($\zeta$) profile.

**Figure 16:**
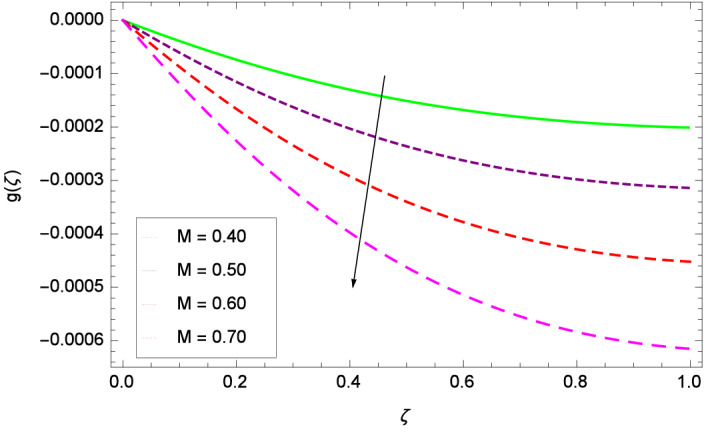
Parameter effect on velocity *g*($\zeta$) profile.

**Figure 17:**
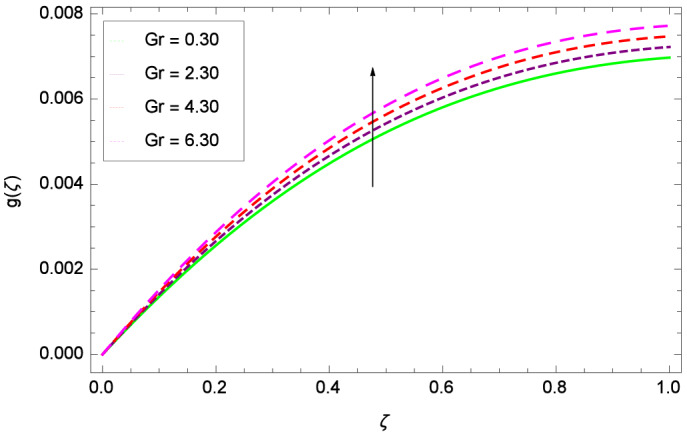
Parameter effect on velocity *g*($\zeta$) profile.

**Figure 18:**
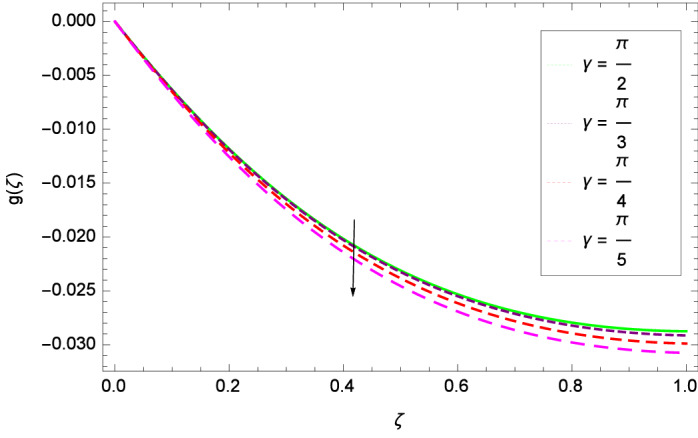
Parameter effect on velocity *g*($\zeta$) profile.

**Figure 19:**
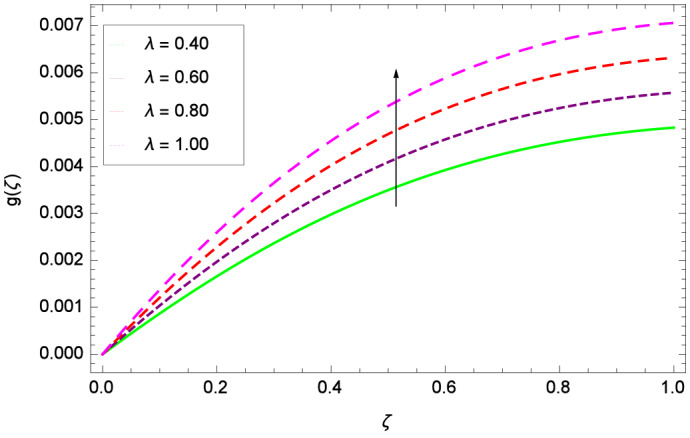
Parameter effect on velocity *g*($\zeta$) profile.

**Figure 20:**
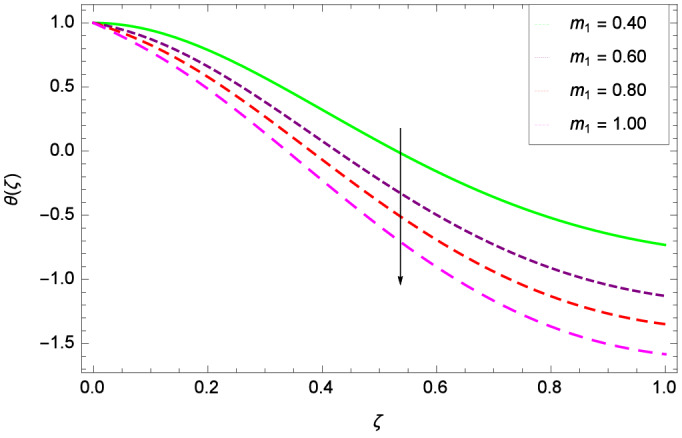
Parameter effect on heat transfer $\theta$($\zeta$) profile.

**Figure 21:**
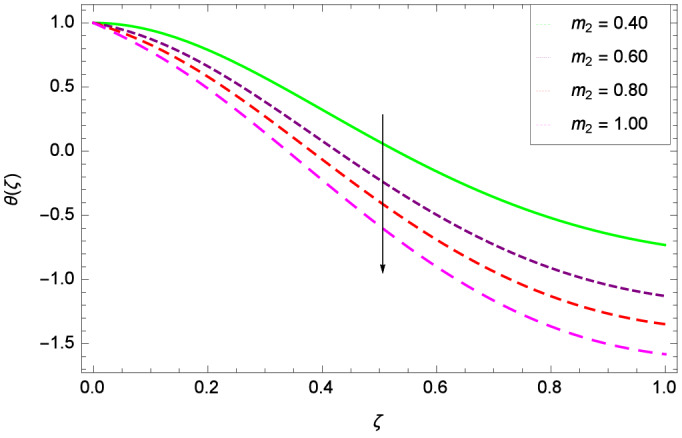
Parameter effect on heat transfer $\theta$($\zeta$) profile.

**Figure 22:**
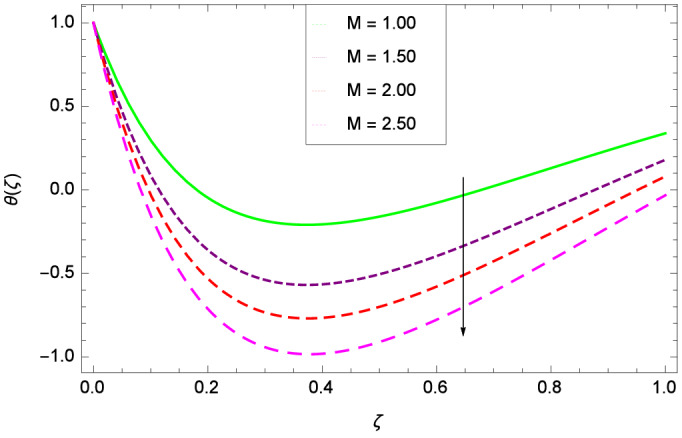
Parameter effect on heat transfer $\theta$($\zeta$) profile.

**Figure 23:**
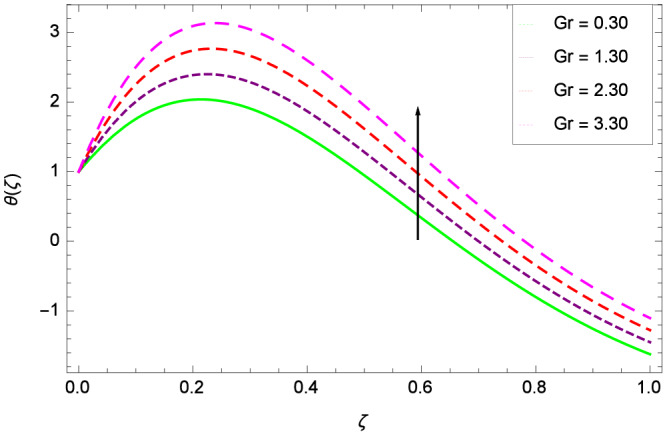
Parameter effect on heat transfer $\theta$($\zeta$) profile.

**Figure 24:**
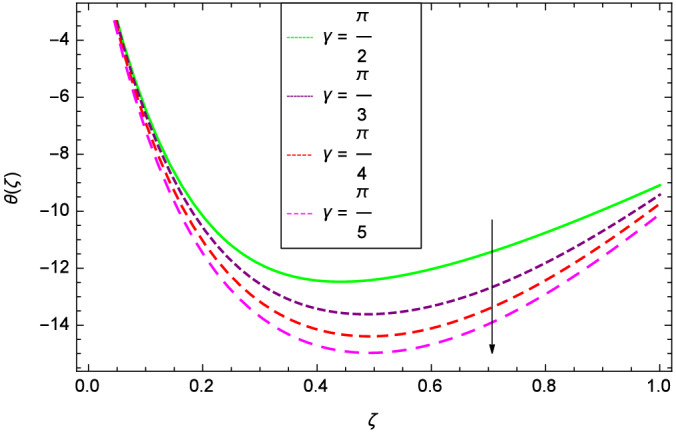
Parameter effect on heat transfer $\theta$($\zeta$) profile.

**Figure 25:**
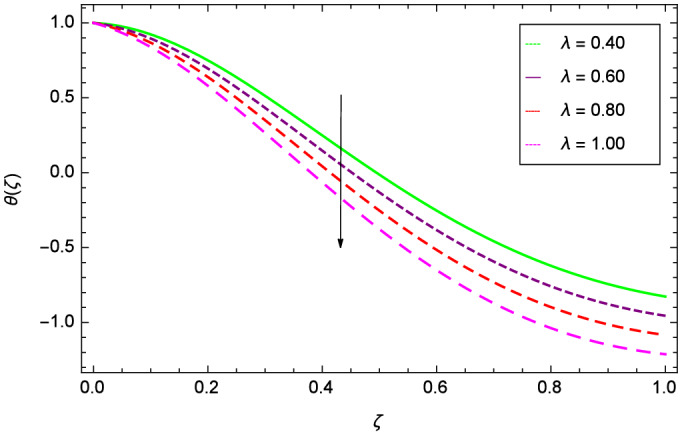
Parameter effect on heat transfer $\theta$($\zeta$) profile.

**Figure 26:**
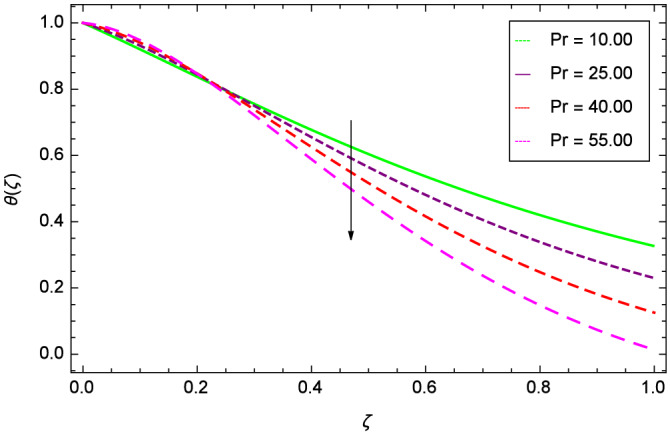
Parameter effect on heat transfer $\theta$($\zeta$) profile.

**Figure 27:**
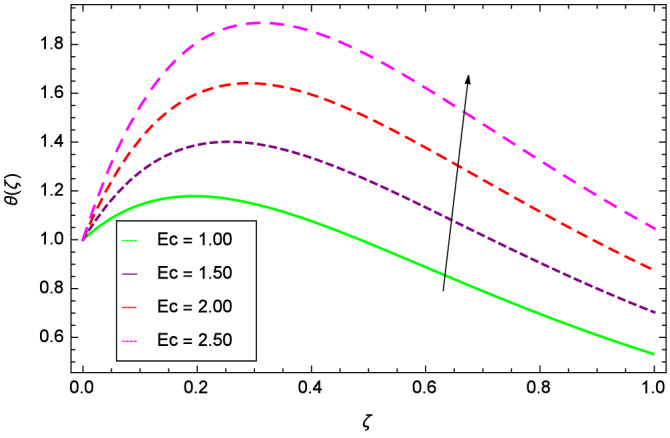
Parameter effect on heat transfer $\theta$($\zeta$) profile.

**Figure 28:**
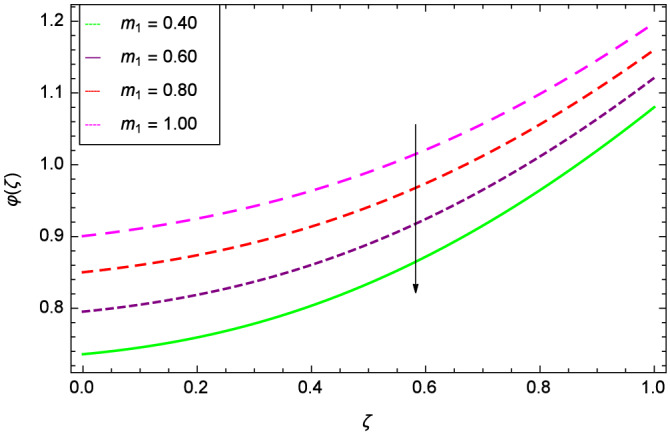
Parameter effect on concentration of homogeneous chemical reactions $\varphi$($\zeta$) profile.

**Figure 29:**
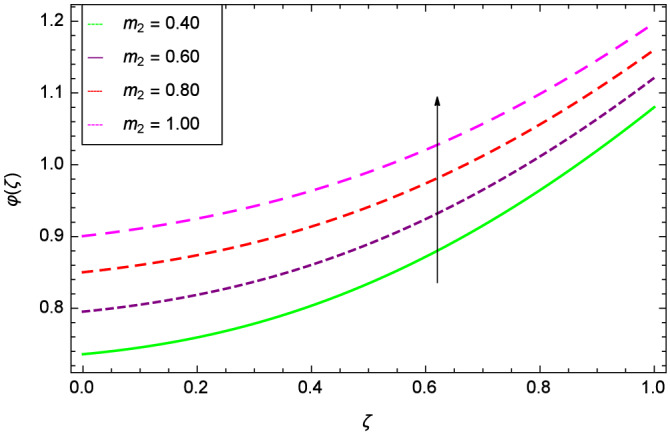
Parameter effect on concentration of homogeneous chemical reactions $\varphi$($\zeta$) profile.

**Figure 30:**
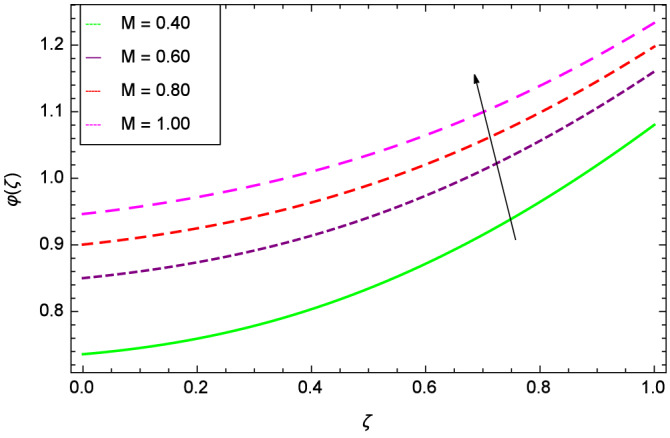
Parameter effect on concentration of homogeneous chemical reactions $\varphi$($\zeta$) profile.

**Figure 31:**
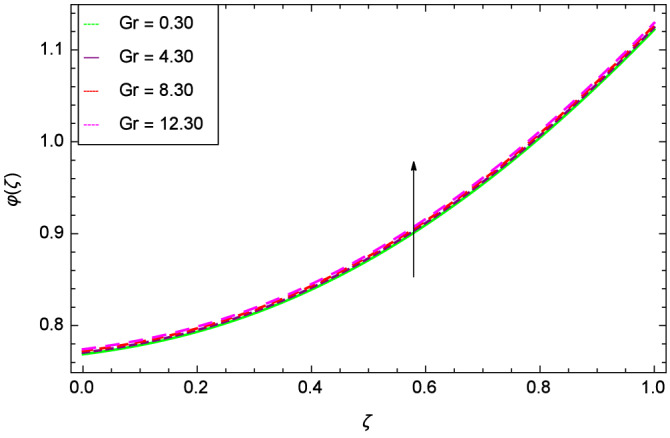
Parameter effect on concentration of homogeneous chemical reactions $\varphi$($\zeta$) profile.

**Figure 32:**
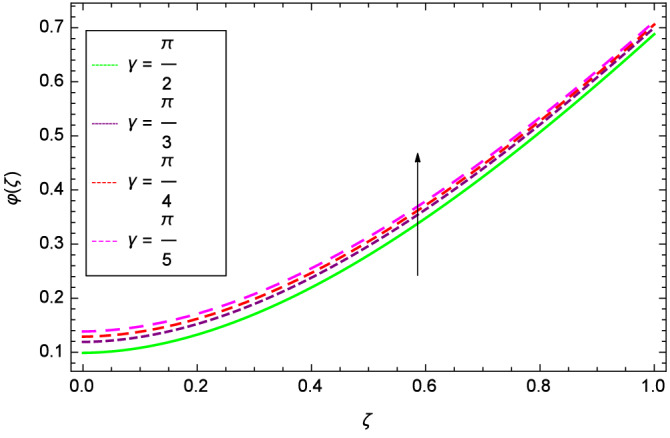
Parameter effect on concentration of homogeneous chemical reactions $\varphi$($\zeta$) profile.

**Figure 33:**
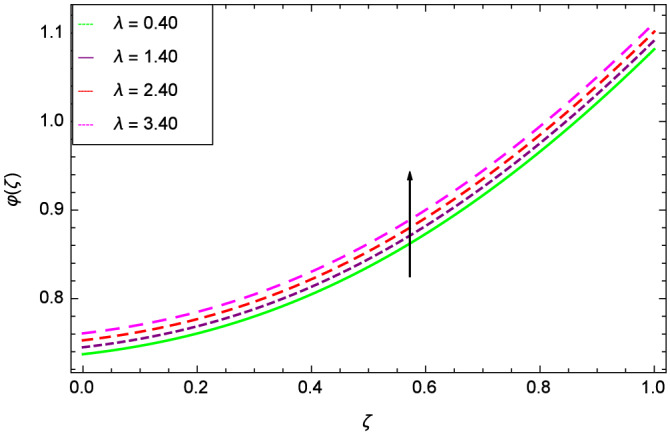
Parameter effect on concentration of homogeneous chemical reactions $\varphi$($\zeta$) profile.

**Figure 34:**
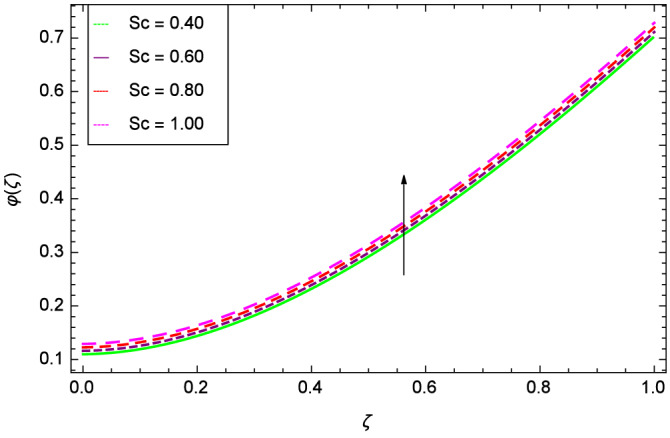
Parameter effect on concentration of homogeneous chemical reactions $\varphi$($\zeta$) profile.

**Figure 35:**
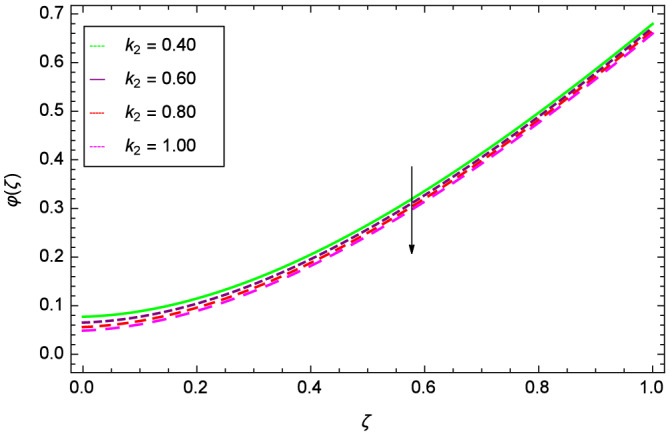
Parameter effect on concentration of homogeneous chemical reactions $\varphi$($\zeta$) profile.

**Figure 36:**
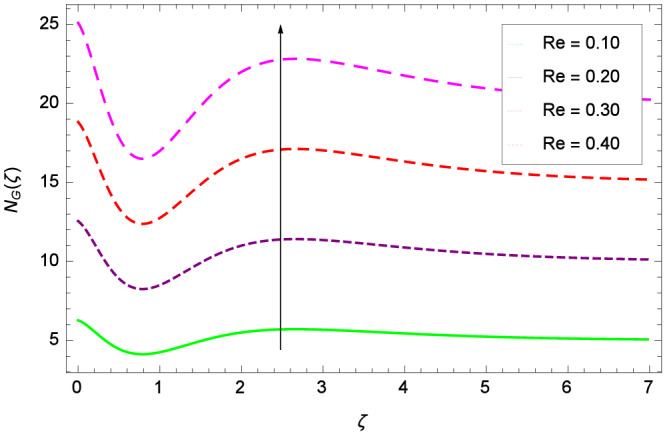
Parameter effect on entropy generation rate $N_{G}$($\zeta$) profile.

**Figure 37:**
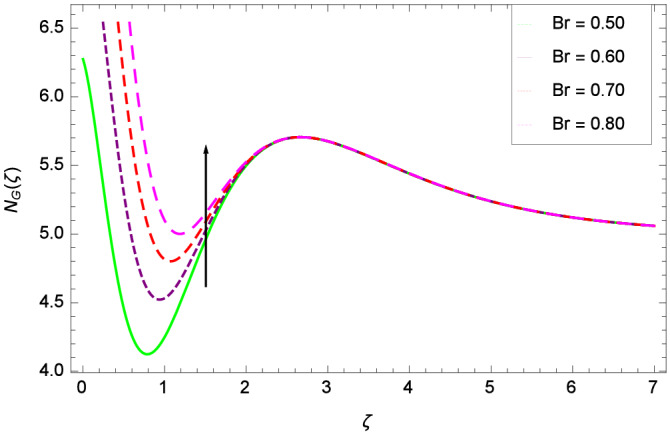
Parameter effect on entropy generation rate $N_{G}$($\zeta$) profile.

**Figure 38:**
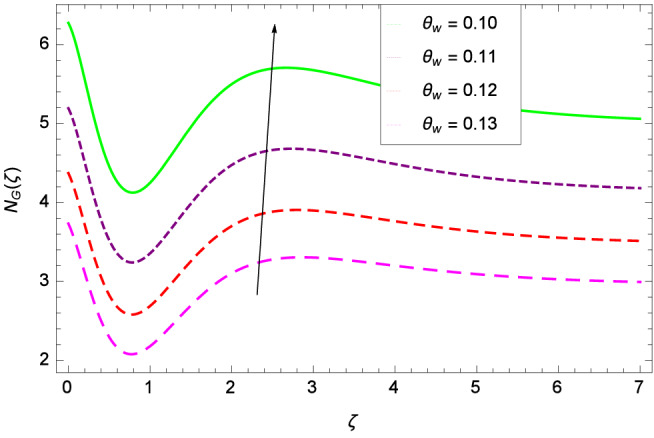
Parameter effect on entropy generation rate $N_{G}$($\zeta$) profile.

**Figure 39:**
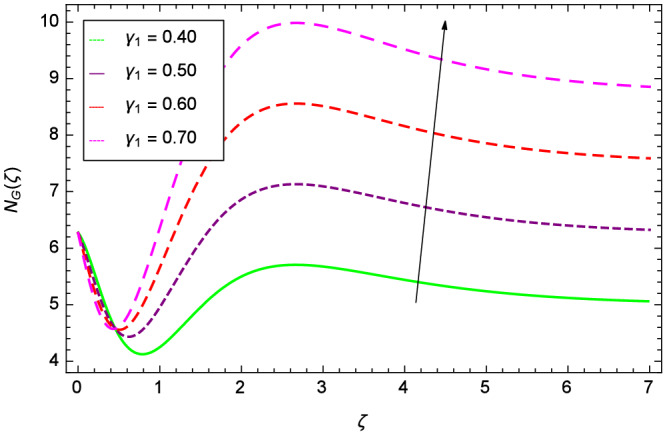
Parameter effect on entropy generation rate $N_{G}$($\zeta$) profile.

## Conclusions

The heat and mass transfer flow of hybrid nanofluid Cu-$\hbox {Fe}_{3}\hbox {O}_{4}$/water with Hall current and ion-slip effects as well as entropy generation is analyzed. The main subject is how the strong and uniform magnetic field convert the two dimensional flow in (*x*, *y*) coordinates into three dimensional flow in (*x*, *y*, *z*) coordinates. Homotopy analysis method is used to compute the solution of the problem. Effects of all the parameters on different profiles are discussed in detail. The authors have intended to investigate the effect of a non-uniform magnetic field in future on the results which has rarely been investigated using an analytical solution. The summary of the results is presented as

1. Axial velocity $f^{\prime }(\zeta )$ decreases with increasing the parameters $m_{1}$, $m_{2}$, *M*, $\gamma$, *Pr* and increases with increasing the parameters *Gr*, $\lambda$, *Ec*.
2. Transverse velocity *g*($\zeta$) decreases with increasing the parameters $m_{1}$, $m_{2}$, *M*, $\gamma$ and increases with increasing the parameters *Gr*, $\lambda$.
3. Temperature decreases with increasing the parameters $m_{1}$, $m_{2}$, *M*, $\gamma$, *Pr*, $\lambda$ and increases with increasing the parameters *Gr*, *Ec*.
4. Concentration of homogeneous chemical reactions decreases with increasing the parameters $m_{1}$, heterogeneous chemical reactions $k_{2}$ and increases with increasing the parameter $m_{2}$, *M*, *Gr*, $\gamma$, $\lambda$, *Sc*.
5. Entropy generation increases with increasing the parameters *Re*, *Br*, $\theta _{w}$ and $\gamma _{1}$.
6. Validation of the present study solution has been given in Table 3.

## Data Availability

All the relevant material is available.

## List of symbols

$$m_{1}$$: Hall parameter
$m_{2}$: Ion-slip parameter
*Gr*: Thermal Grashof number
*Sc*: Schmidt number
*Br*: Brinkman number
*M*: Magnetic field parameter
$$g_{1}$$: Gravitational acceleration
*Pr*: Prandtl number
$$E^{\prime \prime \prime }_{gen}$$: Entropy generation
$E^{\prime \prime \prime }_{0}$: Characteristic entropy generation
*R*: Ideal gas constant
*Re*: Reynolds number
*k*: Thermal diffusivity ($$\hbox {m}^{2}\,\hbox {s}^{-1}$$)
$k_{c}$, $k_{s}$: Rate constants of chemical reactions
$k_{1}$: Homogeneous chemical reactions parameter
$k_{2}$: Heterogeneous chemical reactions parameter
*e*: Electron
$n_{a}$: Neutral particle density
$n_{e}$: Electron number density
$F_{r}$: Friction coefficient between ions and neutral particles
*t*: Time (s)
*x*: *x*-Axis coordinate (m)
*y*: *y*-Axis coordinate (m)
*z*: *z*-Axis coordinate (m)
$U_{\infty }$: Free stream velocity
$$u_{\infty }$$: External velocity
*u*: Velocity component along *x*-axis ($\hbox {m s}^{-1}$)
*v*: Velocity component along *y*-axis ($\hbox {m s}^{-1}$)
*w*: Velocity component along *z*-axis ($\hbox {m s}^{-1}$)
*T*: Temperature (K)
$T_{\infty }$: Ambient fluid temperature (K)
*P*: Pressure ($$\hbox {kg m}^{-1}\,\hbox {s}^{-2}$$)
$c_{p}$: Specific heat at constant pressure ($\hbox {J kg}^{-1}\,\hbox {K}^{-1}$)
*D*: Diffusivity (K)
*B*, *C*: Chemical species
*b* and *c*: Concentrations of *chemical species B* and *C* respectively
*s*: Solid nanoparticles
$f^{\prime }$($\zeta$): Dimensionless axial velocity
*g*($\zeta$): Dimensionless transverse velocity
**B**: Magnetic induction
$B_{0}$: Applied magnetic field strength
$C_{f_{x}}$: Local dimensionless skin friction coefficient in *x*-direction
$C_{g_{z}}$: Local dimensionless skin friction in *z*-direction coefficient
$Nu_{x}$: Local Nusselt number
$Re_{x}$: Local Reynolds number
$q_{w}$: Local wall heat flux (J m$^{-2}$ s$^{-1}$)
$N_{G}$: Dimensionless entropy generation

## Greek symbols

$$\omega _{e}$$: Electron frequency
$$\sigma$$: Electrical conductivity
$\sigma _{1}$: Dimensionless timing parameter
$\lambda$: Stretching/Shrinking parameter
$\psi$: Physical stream function ($\hbox {m}^{2}\,\hbox {s}^{-1}$)
$\beta _{T}$: Thermal expansion coefficient
$$\zeta$$: Similarity variable
$\varphi(\zeta)$: Concentration of homogeneous chemical reactions
$\varphi _{1}(\zeta)$: Concentration of heterogeneous chemical reactions
$\phi$: Total volume fraction of nanoparticles
$\phi _{1}$: Volume fraction of magnetite
$\phi _{2}$: Volume fraction of copper
$\Phi _{i}$, *i* = 1, 2, 3, 4, 5: Dimensionless hybrid nanofluid constants
$$\theta$$($$\zeta$$): Dimensionless temperature
$\theta _{w}$: Temperature difference parameter
$$\nu_{f}$$: Kinematic viscosity ($$\hbox {m}^{2}\,\hbox {s}^{-1}$$)
$$\mu_{hnf}$$: Dynamic viscosity ($$\hbox {kg m}^{-1}\,\hbox {s}^{-1}$$)
$$\rho_{hnf}$$: Density ($$\hbox {kg m}^{-3}$$)
$\tau _{e}$: Electron collision time
$\tau _{w_{x}}$: Surface shear stress in *x*-direction ($\hbox {kg m}^{-1}\,\hbox {s}^{-2}$)
$\tau _{w_{z}}$: Surface shear stress in *z*-direction ($\hbox {kg m}^{-1}\,\hbox {s}^{-2}$)
$\gamma$: Leading edge accretion/ablation parameter
$\gamma _{1}$: Diffusion constant parameter due to nanoparticles concentration

## Subscripts

f: Base fluid
hnf: Hybrid nanofluid
w: Fluid properties at wall
$\infty$: Fluid properties at ambient condition

## Superscripts

$${\prime }$$: Differentiation w. r. t. $$\zeta$$
